# Crystal structures of two nickel(II) macrocyclic salts: (5,7,7,12,12,14-hexa­methyl-1,4,8,11-tetra­aza­cyclo­tetra­deca­ne)nickel(II) bis­(perchlorate) monohydrate and (5,7,7,12,12,14-hexa­methyl-1,4,8,11-tetra­aza­cyclo­tetra­deca­ne)nickel(II) dibromide trihydrate

**DOI:** 10.1107/S2056989019002056

**Published:** 2019-02-08

**Authors:** Peter W. R. Corfield, Virgil L. Goedken

**Affiliations:** a Department of Chemistry, Fordham University, 441 East Fordham Road, Bronx, NY 10458, USA; b Department of Chemistry, The Ohio State University, Columbus, OH 43210, USA

**Keywords:** crystal structure, nickel, macrocycle, cyclam, diasteriomers

## Abstract

The crystal structure of the Ni-14 macrocycle salt, (5,7,7,12,12,14-hexa­methyl-1,4,8,11-tetra­aza­cyclo­tetra­deca­ne)nickel(II) bis­(perchlorate) hemihydrate, [Ni(C_16_H_36_N_4_)]_2_(ClO_4_)_4_·H_2_O, contains two different diasteriomeric macrocyclic cations in the asymmetric unit, one with two NH protons on each side of the cation (**Ia**), and the other with all four NH protons on the same side (**Ib**). The crystal structure of the bromide trihydrate salt of the same Ni-14 macrocyclic cation, namely (5,7,7,12,12,14-hexa­methyl-1,4,8,11-tetra­aza­cyclo­tetra­deca­ne)nickel(II) dibromide trihydrate, [Ni(C_16_H_36_N_4_)]Br_2_·3H_2_O (**II**), contains only the same diastereomer as **Ib**, with the four N—H bonds on the same side. The geometry around the Ni atom differs slightly between the two diastereomeric cations, as the mean Ni—N distance in **Ia** is 1.952 (2) Å, while that for **Ib** and **II** is 1.928 (2) Å. The hexa­methyl substitution in all three macrocyclic cations has the two dimethyl-substituted C atoms *cis* to one another, different from the *trans* 5,5,7,12,12,14-hexa­methyl Ni-14 cations found in all but one of the many published crystal structures of hexa­methyl Ni-14 macrocycles. In each of the two crystal structures, the anions, water mol­ecules, and N—H protons of the macrocyclic cations form extensive hydrogen-bonded zigzag chains propagating along [001] in **I** and [010] in **II**.

## Abstract 2

The crystal structure of the perchlorate salt of a *cis*-hexa­methyl-substituted Ni-14 macrocycle contains two diastereomeric macrocyclic cations in the asymmetric unit, one with two NH protons on each side of the cation, and the other with all four NH protons on the same side. The latter diastereomer is also found in the crystal structure of a bromide trihydrate salt of the same Ni-14 macrocycle.

## Chemical context   

Reports of the formation of cyclic Schiff base–amine complexes of Ni by condensation of acetone with tris­(ethyl­enedi­amine)­nickel(II) salts and their reduction to 14-membered macrocyclic tetra­amine complexes (Curtis, 1960 ▸, 1964 ▸) led to extensive research on these and similar complexes in the 1960 ▸s and 1970s in the hope of using such metal-template reactions in chemical synthesis and of understanding the role of macrocyclic ligands in metalloproteins such as hemoglobin. Their chemical inertness enables chemical reactions of the ligand without losing stereochemistry of the N atoms (Busch, 1978 ▸) and allows characterization of numerous possible isomers (Warner & Busch, 1969 ▸). Crystal structures of isomers of the macrocyclic nickel complexes continue to appear (e.g. Shi *et al.*, 2010 ▸; Curtis *et al.*, 2016 ▸). The major product of the condensation referred to above is a 5,5,7,12,12,14-hexa­methyl-1,4,8,11,tetraaza­cyclo­tetra­deca-4,14-dienenickel(II) ion, where the dimethyl-substituted C atoms are *trans* to each other, and most chemical and structural studies have been concerned with these compounds and their oxidized or reduced species. The 5,7,7,12,12,14-hexa­methyl-1,4,8,11,tetra­aza­cyclo­tetra­deca­nenickel(II) com­pounds presented here, abbreviated as *cis*-[Nime_6_cyclam]^2+^, where the dimethyl-substituted C atoms are *cis* to one another, are derived from the minor product of the condensation, which has received less attention.
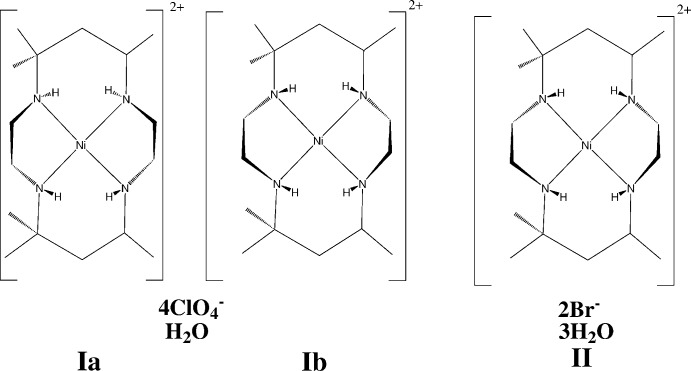


## Structural commentary   

**Figure 1:**
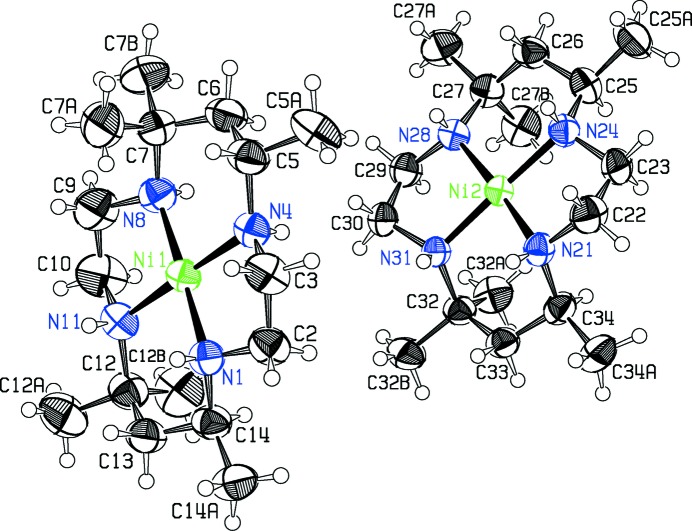
The [Nime_6_cyclam]^2+^ cations in the asymmetric unit of the double salt **I**. Displacement ellipsoids are drawn at the 50% probability level. The cation centered on Ni1 is structure **Ia** in the text, and the other cation is **Ib**.

Compound **I** crystallizes as a double salt, containing two independent *cis*-[Nime_6_cyclam]^2+^ cations, with structures **Ia** and **Ib** in the scheme, four ClO_4_^−^ anions, and one water of hydration in the asymmetric unit. Compound **II** crystallizes as a trihydrate built from *cis*-[Nime_6_cyclam]^2+^ cations, with structure **II** in the scheme, two Br^−^ anions and three water mol­ecules. The configurations of cations **Ib** and **II** are the same. Figs. 1 ▸–3 ▸ ▸ display the cations, anions, and packing diagram for compound **I**, while Figs. 4 ▸–6 ▸ ▸ give the cation, packing diagram and proposed hydrogen-bonding network for **II**.

In each cation, the nickel atom is in square-planar coord­in­ation to the macrocycle, with the Ni and four N atoms in a close to planar arrangement. All six-membered chelate rings are in the chair form, and all singly substituted methyl groups are in the equatorial position. In the reference mol­ecule for **Ia** there are two NH atoms above and two below the N_4_ plane, designated as *uudd*, in an *RRSS* configuration, whereas cations **Ib** and **II** are diastereomers of **Ia**, with all four NH atoms lying on the same side of the mol­ecule, *uuuu*, and the N atoms in an *RSRS* configuration. Cation **Ia** is roughly planar in overall shape, whereas the N—H geometry in **Ib** and **II** makes the cations in these structures more bowl shaped. The configurational differences at N appear to affect the Ni—N bond lengths slightly: the mean Ni—N distance in **Ia** is 1.952 (2) Å while that for **Ib** and **II** is 1.928 (2) Å.

The conformations of the five-membered chelate rings in the reference cations shown in the scheme are λ on the left and δ on the right for **Ia**, and λ on the right and δ on the left for **Ib** and **II**. (Mirror-related cations are present in both crystals.) The twists of these five-membered rings necessarily differentiate between the top and bottom six-membered chelate rings in **Ib** and **II**, whereas this is not the case in **Ia**. In diastereomers **Ib** and **II**, the top plane (N4, C5, C7, N8) is bent at a less steep angle to the NiN_4_ coordination plane than the bottom plane (N11, C12, C14, N1) (add 20 to atom numbers for structure **Ib**) and the outer C atoms C6 and C13 are at widely different distances from the NiN_4_ plane. Thus in **Ib** and **II**, the angles between the NiN_4_ plane and the N_2_C_2_ plane of the top chelate ring are 29.6 (1) and 31.7 (3)°, respectively, while corresponding angles for the bottom rings are 52.7 (2) and 57.1 (2)°. The top outer carbon C6 is 0.317 (6) Å from the N_4_ plane in **Ib** and 0.407 (10) Å in **II**, while the corresponding distances for the bottom outer atom C13 are respectively 1.176 (5) and 1.314 (11) Å. The Ni coordination geometry reflects this difference between the top and bottom of the mol­ecule, with the top N4—Ni—N8 angle opened out to 94.58 (12)° in **Ib** and 94.79 (19)° in **II**, compared with bottom angles N1—Ni—N11 of 88.72 (12) and 87.73 (19)°, respectively. The five-membered chelate ring angles at the Ni atom, N1—Ni—N4 and N8—Ni—N11, average 88.48 (16)° in these two structures.

Mol­ecule **Ia** is less-buckled, with angles between the N_4_ plane and central planes of the chelate chairs more nearly equal, at 27.6 (2)° for the top chair and 31.9 (2)° for the bottom, and outer C atom distances from the N_4_ plane of 0.250 (6) Å for C6 at the top, and −0.389 (6) for C13 at the bottom. The Ni coordination plane is more nearly symmetrical, with six-membered chelate angles N4—Ni1—N8 of 93.49 (14)° (top) and N1—Ni1—N11 of 92.88 (13)° (bottom), and five-membered chelate angles averaging 86.87 (13)°, somewhat smaller than for **Ib** and **II**.

In both of the **Ib** and **II** cations, hydrogen bonding of an anion or of a solvent mol­ecule brings an O atom close to the axial direction of the Ni atom on the same side of the cation as the four NH bonds, though at distances too long to be regarded as due to Ni—O bonding. In **Ib**, perchlorate atom O31 is at 2.799 (3) Å from atom Ni2, while in **II**, water mol­ecule O1 is at 2.863 (10) Å from the Ni atom.

## Supra­molecular features   

Details of hydrogen bonding are given in Tables 1 ▸ and 2 ▸. The N—H bonds in all three cations form hydrogen bonds; to water or perchlorate O atoms in **I**, and to water O atoms or Br^−^ ions in **II**.

**Figure 3:**
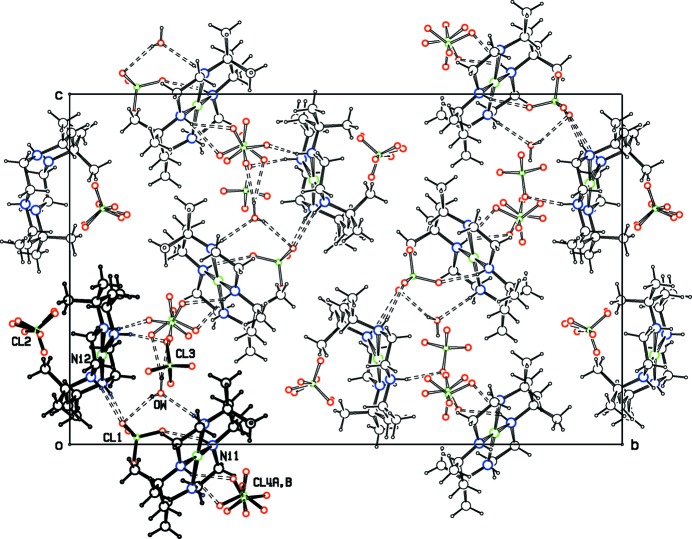
Projection down the *a* axis for the double salt, **I**, showing the hydrogen-bonded network extending along the *c-*axis direction. Ions and the water mol­ecule in the asymmetric unit are in bold.

**Figure 4:**
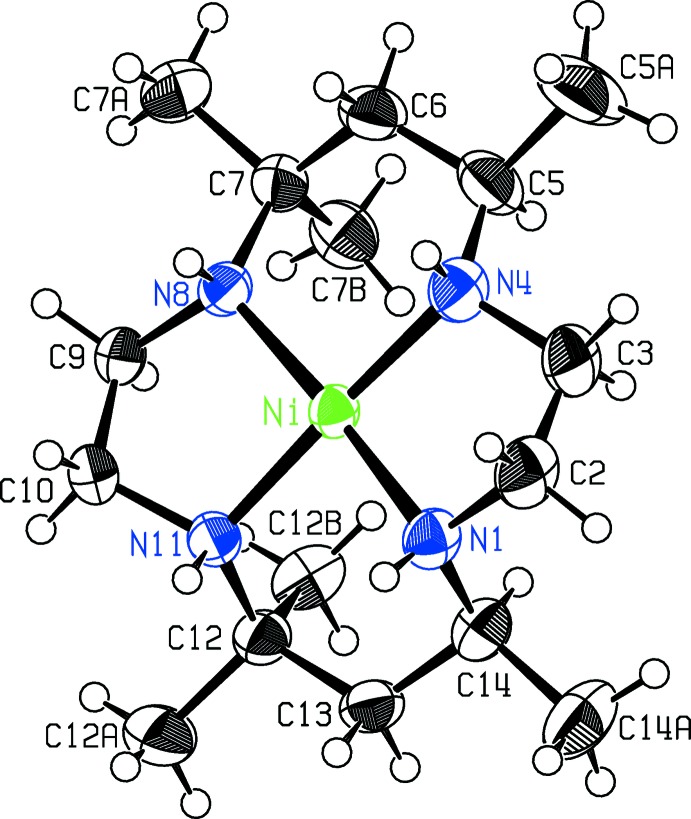
The [Nime_6_cyclam]^2+^cation in the macrocycle bromide salt **II**. Displacement ellipsoids are at the 50% probability level.

In the double salt **I**, hydrogen bonding between the cations, the four perchlorate ions ClO_4_^−^(1)–ClO_4_^−^(4) and the water mol­ecule form a one-dimensional network extending along the *c*-axis direction, as shown in Fig. 3 ▸. Three of the four O atoms in the relatively ordered ClO_4_^−^(1) anion link the two reference mol­ecules together by N—H⋯O hydrogen bonds. Neither of the alternative orientations for ClO_4_^−^(2) form any N—H⋯O or O—H⋯O H bonds. These disordered ions lie in a hydro­phobic cavity in the crystal structure, and may be held in position by C—H⋯O bonds. The relatively ordered ion ClO_4_^−^(3) is tethered by only one hydrogen bond, while each orientation for disordered ClO_4_^−^(4) is hydrogen bonded to the water mol­ecule and to either one or two N—H groups of the cations. The water mol­ecule is well stabilized in its position by three separate hydrogen bonds.

**Figure 6:**
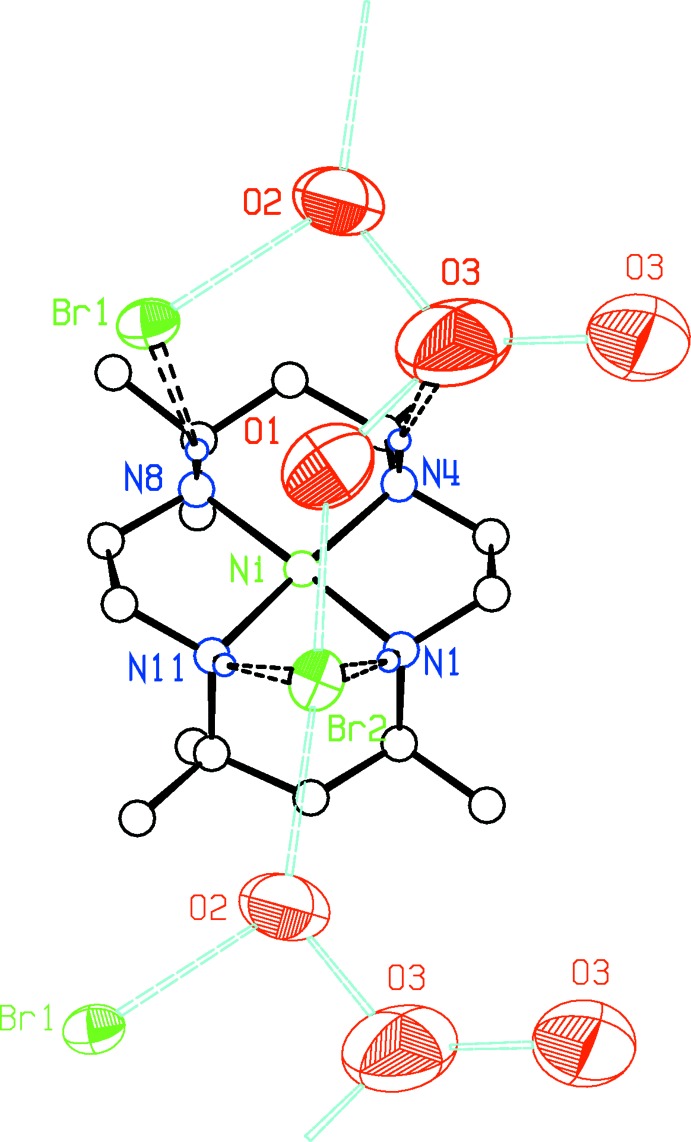
Details of the proposed hydrogen-bond network for the macrocycle bromide salt **II**. Displacement ellipsoids are at the 50% probability level, with anions and solvent in the asymmetric unit drawn in bold. Bromide ions are green, and water O atoms red. Putative hydrogen bonds involving water mol­ecules for which protons were not found are in cyan while other hydrogen bonds are black. Water O atoms and Br1 at the top of the figure are related to the corresponding atoms at the bottom *via* the translation vector (0, ½, −½).

The cyclam cation in **II** forms hydrogen bonds to the Br^−^ ions *via* N1—H1, N8—H8 and N11—H11, while N4—H4 hydrogen-bonds to water mol­ecule O3. O3 appears to form rather short hydrogen bonds with water mol­ecules O1 and O2, as well as with O3 rotated by the crystallographic twofold axis at *x* = *y* = 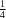, with respective O⋯O distances of 2.671 (11), 2.635 (10) and 2.638 (12) Å. Exact details of the hydrogen-bonding network are not clear, as none of the water H atoms could be located with assurance (see *Refinement* section) However, distances O1⋯Br2 = 3.341 (9) Å, O2 ⋯ Br1 = 3.347 (9) Å, and O2 ⋯ Br2(*x*, *y* − 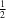, *z* + 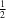) = 3.332 (8) Å are consistent with water–bromide ion hydrogen bonding, which would give rise to the hydrogen-bonding network suggested in Fig. 6 ▸. Short ribbons along the (0, 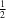, −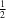) direction linked to each other *via* presumed O3⋯O3 hydrogen bonds across the twofold axes lead to the formation of extended zigzag chains along the *b-*axis direction.

The shortest (C)H⋯(C)H distances are 2.61 Å in **I**, between H27*F* and H32*B*(*x*, 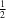 − *y*, 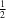 + *z*), with just four other contacts less than 2.70, and 2.47 Å in **II**, between H9*B* and H12*E*(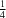 − *x*, −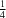 + *y*, −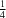 + *z*), with five other contacts less than 2.70 Å.

## Database survey   

A search in the Cambridge Structural Database (CSD, Version of 2017; Groom *et al.*, 2016 ▸) for *cis*-[Nime_6_cyclam]^2+^ structures produced only one hit (TICCOX; Wang *et al.*, 1996 ▸). This structure has a configuration with all NH atoms on the same side of the mol­ecule, or *uuuu*, with a configuration the same as that of the structures **Ib** and **II** in the present work. The sole other *cis*-cyclam structure of any kind has Cu as the chelated metal ion (HMTZCP; Ochiai *et al.*, 1978 ▸), with a configuration the same as that of structure **Ia**.

Of 38 3D *trans*-[Nime_6_cyclam]^2+^structures found in the CSD, 26 have the NH configuration *uudd* of cation **Ia** in the present work, five have a *udud* configuration, and five have the NH configuration *uuuu* (or equivalently *dddd*), but with λλ or δδ conformations for the five-membered chelate rings, different from the conformations of **Ib** and **II** in the present work. In these 36 structures, there need be no difference between the geometries of the six-membered chelate rings, and indeed, minus a few exceptions, both N—Ni—N six-membered ring chelate angles are identical, with a mean of 93.2 (4)°. The last two *trans* structures [LIFYEG (Ou *et al.*, 2013 ▸), NIBTET (Curtis *et al.*, 1973 ▸)] have cations with the same conformation as in **Ib** and **II**, and with the same differentiation in six-membered ring N—Ni—N chelate angles as in the present work.

A search for structures where Ni^2+^ is coordinated solely by the unsubstituted cyclam ligand gave 20 hits. Of these, one had the *RRRR* configuration, or *udud*, with alternate NH atoms pointing upwards and downwards, while 19 had the *RRSS* configuration, or *uudd*, as in the present **Ia** structure, the more stable isomer according to Bosnich *et al.* (1965 ▸). None of these unsubstituted Ni-cyclam structures had the *RSRS* configuration, or *uuuu*, with all NH atoms on the same side of the mol­ecule, as in the present **Ib** and **II** structures. Presumably this particular configuration is stabilized by the methyl substituent groups.

## Synthesis and crystallization   

The double salt **I** was prepared in Daryle H. Busch’s laboratories by methods described in Curtis (1967 ▸). The bromide salt **II** was prepared by a solution of **I** in methanol/KBr/HBr, precipitation with ether, and recrystallization from hot aqueous HBr.

## Refinement   

Crystal data, data collection and structure refinement details are summarized in Table 3 ▸.

Data for **I** were collected at The Ohio State University many years ago. As was the custom then, reflection data were stored as *F* values, so that those reflections for which *F* ^2^ values were negative were stored with values of zero. During the preparation of this manuscript, we found that the original absorption correction had been carried out with an incorrect value for the absorption coefficient, μ. While correcting this problem, we converted the reflection data into the *F* ^2^ values used in the final refinements. Thermal parameters for the perchlorate O atoms in **I** are all large, indicating probable positional disorder, common for these anions. After extensive modeling attempts, ClO_4_^−^ ions 1 and 3 were refined with an ordered model, while ClO_4_^−^ ions 2 and 4 were refined in two alternative orientations, with 50% occupancy each and a common Cl atom in ClO_4_^−^(2), and occupancies of 65.0 (8)% and 35.0 (8)% and separate sites for the disordered Cl atoms in ClO_4_^−^(4). Initially, tight restraints on the ClO_4_ geometry were imposed, but these were relaxed during the final refinements. However, it proved useful to impose restraints on the thermal parameters for the O atoms with the Shelx RIGU command, and a DFIX command was used to prevent the too close approach of two O atoms from different perchlorate groups.

Crystal data for compound **II**, the bromide salt, were originally obtained with the same Picker four-circle diffractometer as used for compound **I**. (Three octa­nts merged to give 1916 observations; Gaussian absorption correction applied; *R*_1_ = 0.026 for 1780 observed > 2σ, *R*_2_ = 0.078, NV = 241, GOOF = 0.876, Δρ = −0.42 to +0.60 e Å^−3^.) We recollected data on the same crystal much later with the KappaCCD system at Fordham University to expand the data set and because some of the previous processing details had been lost. Refinements with the two sets of data gave very similar results, with no bond length or inter­ior bond angle differing by more than 2.0σ and average difference 0.7σ. Twinning by reflection about the (001) plane, perpendicular to the polar twofold axis in *Fdd*2, was indicated by the Flack parameter of 0.57 (2) as well as by the low value of 0.030 found for *R*_merg_ if the observed *I*(*hkl*) and *I*(*hk* 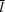) intensities were merged, compared with 0.070 if the calculated intensities for an untwinned crystal were merged.

As noted in the section on *Supra­molecular features*, the water mol­ecules refined to positions rather close to one another. It was necessary to introduce anti-bumping restraints in the *SHELXL* refinements to avoid unreasonably short O⋯O contacts. Difference maps at the end of the least-squares refinements were dominated by features associated with the Br^−^ ions, and were uninformative regarding the positions of H atoms, even when calculated with only low-angle data. Thus, none of the H atoms on the water mol­ecules were located. Potential positions for some water H atoms could be derived from the presumed hydrogen-bonding pattern, but refinements including these atoms were inconclusive. We tried refining the *SHELXL* BASF factor to see if this improved the difference maps, obtaining BASF = 0.58 (2), with negligible changes in the difference map or *R* factors. Hence our final refinements assume equal contributions from each twin component. The close proximity of O3 to the crystallographic twofold axis suggests disorder of at least the H atoms on O3, and the large *U*_eq_ value for O3 suggests probable disorder of the O3 atoms themselves. It was possible to generate two closely positioned sites for O3, but extensive efforts to refine a suitable disordered model for O3 did not improve the *R* factors, nor give more reasonable *U*_eq_ values for the disordered O3 atoms, while difference maps from these refinements did not give any useful information either on water H atoms. In light of these factors, we have not reported a model with a disordered O3 atom.

In both compounds, H atoms on the cation were constrained to idealized positions, with C—H distances of 0.97 Å for the methyl­ene groups, 0.98 Å for the methine CH groups, and 0.96 Å for the methyl groups, while the *U*_eq_ factors for these H atoms were set at 1.2 times the *U*_iso_ of the bonded atoms for methyl­ene and methine groups, and 1.5 times for the methyl groups. All NH atoms were refined, with *U*_eq_ values set at 1.2 times the *U*_iso_ for their bonded N atom in **I** and 1.0 times *U*_iso_ for **II**.

## Supplementary Material

Crystal structure: contains datablock(s) I, II. DOI: 10.1107/S2056989019002056/pk2613sup1.cif

Structure factors: contains datablock(s) I. DOI: 10.1107/S2056989019002056/pk2613Isup2.hkl

Structure factors: contains datablock(s) II. DOI: 10.1107/S2056989019002056/pk2613IIsup3.hkl

CCDC references: 1895686, 1895685

Additional supporting information:  crystallographic information; 3D view; checkCIF report

## Figures and Tables

**Figure 2:**
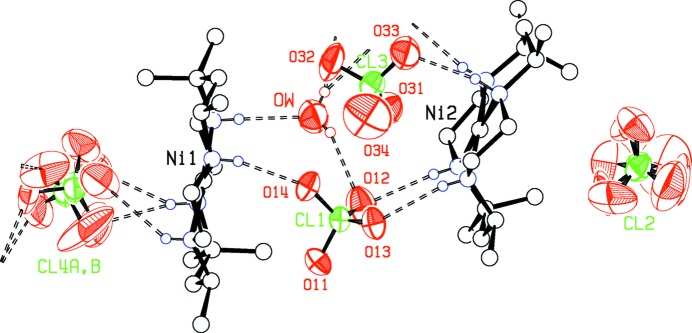
The perchlorate anions and water mol­ecule in the asymmetric unit of double salt **I**, showing their relationship with the cations, and hydrogen bonds formed. The disordered ClO_4_^−^(2) anion does not appear to form any hydrogen bonds. Displacement ellipsoids are drawn at the 50% probability level.

**Figure 5:**
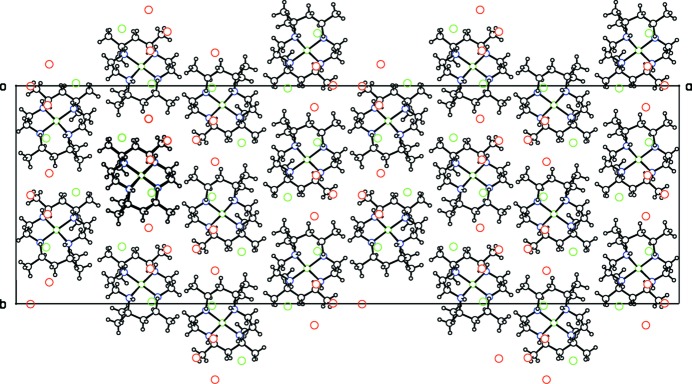
Projection down the *c* axis for the macrocycle bromide salt **II**. The asymmetric unit is in bold. Bromide ions are green, and water mol­ecules red.

**Table 1:** Hydrogen-bond geometry (Å, °) for **I** [Chem scheme1]

*D*—H⋯*A*	*D*—H	H⋯*A*	*D*⋯*A*	*D*—H⋯*A*
N1—H1⋯O44	0.98	2.61	3.279 (9)	126
N4—H4⋯O14	0.98	2.02	2.941 (4)	157
N8—H8⋯O*W*	0.98	1.99	2.965 (5)	175
N11—H11⋯O43	0.98	2.11	3.028 (10)	155
N11—H11⋯O46	0.98	2.45	3.36 (3)	155
N21—H21⋯O13	0.98	2.14	3.093 (4)	165
N24—H24⋯O33	0.98	2.12	3.033 (5)	154
N28—H28⋯O45^i^	0.98	2.30	3.146 (16)	144
N31—H31⋯O12	0.98	2.16	3.083 (4)	156
O*W*—H*W*1⋯O41^i^	0.82 (1)	2.38 (2)	3.162 (11)	162 (5)
O*W*—H*W*1⋯O47^i^	0.82 (1)	2.37 (3)	3.139 (18)	157 (5)
O*W*—H*W*2⋯O12	0.82 (1)	2.45 (3)	3.181 (6)	149 (6)

Symmetry code: (i) 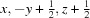.

**Table 2:** Hydrogen-bond geometry (Å, °) for **II** [Chem scheme1]

*D*—H⋯*A*	*D*—H	H⋯*A*	*D*⋯*A*	*D*—H⋯*A*
N1—H1⋯Br2	0.81 (6)	2.66 (6)	3.461 (5)	169 (6)
N4—H4⋯O1	0.80 (6)	2.80 (7)	3.283 (11)	121 (5)
N4—H4⋯O3	0.80 (6)	2.25 (6)	3.008 (13)	159 (6)
N8—H8⋯Br1	0.83 (6)	2.63 (6)	3.444 (5)	164 (5)
N11—H11⋯Br2	0.89 (6)	2.63 (6)	3.466 (4)	159 (5)

**Table 3:** Experimental details

	**I**	**II**
Crystal data		
Chemical formula	[Ni(C_16_H_36_N_4_)]_2_(ClO_4_)_4_·H_2_O	[Ni(C_16_H_36_N_4_)]Br_2_·3H_2_O
*M*_r_	1102.21	557.06
Crystal system, space group	Monoclinic, *P*2_1_/*c*	Orthorhombic, *F* *d* *d*2
Temperature (K)	295	295
*a*, *b*, *c* (Å)	8.906 (4), 29.412 (11), 19.505 (9)	60.3649 (18), 19.8364 (9), 7.9773 (3)
α, β, γ (°)	90, 107.030 (19), 90	90, 90, 90
*V* (Å^3^)	4885 (4)	9552.2 (6)
*Z*	4	16
Radiation type	Cu *K*α	Mo *K*α
μ (mm^−1^)	3.60	4.17
Colour	Orange	Yellow
Crystal size (mm)	0.52 × 0.25 × 0.11	0.37 × 0.15 × 0.10

Data collection		
Diffractometer	Picker 4-circle	Enraf–Nonius KappaCCD
Radiation source	sealed X-ray tube	fine-focus sealed tube
Absorption correction	Gaussian (Busing & Levy, 1957 ▸)	Part of the refinement model (Δ*F*) (*SCALEPACK*; Otwinowski & Minor, 1997 ▸)
*T*_min_, *T*_max_	0.454, 0.686	0.34, 0.67
No. of measured, independent and observed [*I* > 2σ(*I*)] reflections	7450, 6870, 4899	41142, 5382, 4897
*R*_int_	0.060	0.096
θ_max_ (°)	58.4	27.5
(sin θ/λ)_max_ (Å^−1^)	0.552	0.649

Refinement		
*R*[*F* ^2^ > 2σ(*F* ^2^)], *wR*(*F* ^2^), *S*	0.043, 0.109, 1.04	0.035, 0.085, 1.04
No. of reflections	6870	5382
No. of parameters	686	253
No. of restraints	184	4
H-atom treatment	H atoms treated by a mixture of independent and constrained refinement	H atoms treated by a mixture of independent and constrained refinement
Δρ_max_, Δρ_min_ (e Å^−3^)	0.30, −0.31	0.88, −0.56

Data reduction followed procedures in Corfield *et al.* (1973 ▸). Structure solution was by the heavy-atom method with local programs. Computer programs: Corfield & Gainsford (1972 ▸), *KappaCCD Server Software* (Nonius, 1997 ▸), *DENZO* and *SCALEPACK* (Otwinowski & Minor, 1997 ▸), *SHELXL2017* (Sheldrick, 2015 ▸), *ORTEPIII* (Burnett & Johnson, 1996 ▸), *ORTEP-3 for Windows* (Farrugia, 2012 ▸) and *publCIF* (Westrip, 2010 ▸).

## supplementary crystallographic information

### (5,7,7,12,12,14-Hexamethyl-1,4,8,11-tetraazacyclotetradecane)nickel(II) bis(perchlorate) hemihydrate (I) Crystal data

**Table:**

[Ni(C_16_H_36_N_4_)]_2_(ClO_4_)_4_·H_2_O	*F*(000) = 2328
*M**_r_* = 1102.21	*D*_x_ = 1.499 Mg m^−^^3^*D*_m_ = 1.49 Mg m^−^^3^*D*_m_ measured by flotation in chloroform/carbon tetrachloride mixtures
Monoclinic, *P*2_1_/*c*	Cu *K*α radiation, λ = 1.5418 Å
*a* = 8.906 (4) Å	Cell parameters from 28 reflections
*b* = 29.412 (11) Å	θ = 5.2–28.2°
*c* = 19.505 (9) Å	µ = 3.60 mm^−^^1^
β = 107.030 (19)°	*T* = 295 K
*V* = 4885 (4) Å^3^	Needle, orange
*Z* = 4	0.52 × 0.25 × 0.11 mm

### (5,7,7,12,12,14-Hexamethyl-1,4,8,11-tetraazacyclotetradecane)nickel(II) bis(perchlorate) hemihydrate (I) Data collection

**Table:**

Picker 4-circle diffractometer	4899 reflections with *I* > 2σ(*I*)
Radiation source: sealed X-ray tube	*R*_int_ = 0.060
Oriented graphite 200 reflection monochromator	θ_max_ = 58.4°, θ_min_ = 2.8°
θ/2θ scans	*h* = 0→9
Absorption correction: gaussian (Busing & Levy, 1957)	*k* = 0→32
*T*_min_ = 0.454, *T*_max_ = 0.686	*l* = −21→21
7450 measured reflections	3 standard reflections every 200 reflections
6870 independent reflections	intensity decay: +2(5)

### (5,7,7,12,12,14-Hexamethyl-1,4,8,11-tetraazacyclotetradecane)nickel(II) bis(perchlorate) hemihydrate (I) Refinement

**Table:**

Refinement on *F*^2^	Primary atom site location: heavy-atom method
Least-squares matrix: full	Secondary atom site location: difference Fourier map
*R*[*F*^2^ > 2σ(*F*^2^)] = 0.043	Hydrogen site location: mixed
*wR*(*F*^2^) = 0.109	H atoms treated by a mixture of independent and constrained refinement
*S* = 1.04	*w* = 1/[σ^2^(*F*_o_^2^) + (0.016*P*)^2^ + 2.*P*] where *P* = (*F*_o_^2^ + 2*F*_c_^2^)/3
6870 reflections	(Δ/σ)_max_ = 0.001
686 parameters	Δρ_max_ = 0.30 e Å^−^^3^
184 restraints	Δρ_min_ = −0.30 e Å^−^^3^

### (5,7,7,12,12,14-Hexamethyl-1,4,8,11-tetraazacyclotetradecane)nickel(II) bis(perchlorate) hemihydrate (I) Special details

**Table:**

Geometry. All esds (except the esd in the dihedral angle between two l.s. planes) are estimated using the full covariance matrix. The cell esds are taken into account individually in the estimation of esds in distances, angles and torsion angles; correlations between esds in cell parameters are only used when they are defined by crystal symmetry. An approximate (isotropic) treatment of cell esds is used for estimating esds involving l.s. planes.

### (5,7,7,12,12,14-Hexamethyl-1,4,8,11-tetraazacyclotetradecane)nickel(II) bis(perchlorate) hemihydrate (I) Fractional atomic coordinates and isotropic or equivalent isotropic displacement parameters (Å^2^)

**Table:**

	*x*	*y*	*z*	*U*_iso_*/*U*_eq_	Occ. (<1)
Ni1	0.33013 (7)	0.23109 (2)	−0.03275 (3)	0.04565 (18)	
N1	0.1761 (4)	0.22148 (10)	−0.12620 (15)	0.0485 (8)	
H1	0.197135	0.244899	−0.157932	0.058*	
C2	0.0171 (5)	0.23125 (15)	−0.1204 (2)	0.0625 (11)	
H2A	−0.025056	0.204666	−0.103052	0.075*	
H2B	−0.053194	0.239506	−0.166885	0.075*	
C3	0.0328 (5)	0.26965 (15)	−0.0690 (2)	0.0666 (12)	
H3A	0.058301	0.297432	−0.090027	0.080*	
H3B	−0.065282	0.274219	−0.057861	0.080*	
N4	0.1597 (4)	0.25839 (10)	−0.00289 (15)	0.0486 (8)	
H4	0.117301	0.233348	0.018922	0.058*	
C5	0.1831 (5)	0.29636 (14)	0.0503 (2)	0.0633 (12)	
H5	0.204365	0.324159	0.027016	0.076*	
C6	0.3218 (5)	0.28734 (14)	0.1143 (2)	0.0664 (12)	
H6A	0.304739	0.258561	0.135098	0.080*	
H6B	0.324884	0.310785	0.149625	0.080*	
C7	0.4812 (5)	0.28563 (15)	0.1010 (2)	0.0683 (12)	
N8	0.4865 (4)	0.24318 (11)	0.05883 (16)	0.0550 (8)	
H8	0.474060	0.218272	0.090082	0.066*	
C9	0.6423 (5)	0.23559 (19)	0.0486 (2)	0.0823 (15)	
H9A	0.720540	0.231303	0.094621	0.099*	
H9B	0.672292	0.261661	0.025142	0.099*	
C10	0.6320 (6)	0.19438 (18)	0.0036 (3)	0.0879 (16)	
H10A	0.614938	0.167630	0.029429	0.105*	
H10B	0.728654	0.190295	−0.009098	0.105*	
N11	0.4978 (4)	0.20118 (10)	−0.06199 (16)	0.0533 (8)	
H11	0.534956	0.224885	−0.088376	0.064*	
C12	0.4711 (5)	0.16056 (14)	−0.1118 (2)	0.0636 (11)	
C13	0.3333 (5)	0.17096 (15)	−0.1773 (2)	0.0690 (12)	
H13A	0.356791	0.198604	−0.199267	0.083*	
H13B	0.324394	0.146555	−0.211698	0.083*	
C14	0.1776 (5)	0.17674 (13)	−0.1638 (2)	0.0592 (11)	
H14	0.165234	0.152268	−0.131766	0.071*	
C5A	0.0356 (6)	0.30423 (17)	0.0732 (3)	0.0869 (15)	
H5A1	−0.049541	0.312685	0.032148	0.130*	
H5A2	0.054457	0.328153	0.108138	0.130*	
H5A3	0.008990	0.276807	0.093638	0.130*	
C7A	0.5102 (7)	0.32751 (16)	0.0600 (3)	0.0999 (18)	
H7A1	0.618224	0.328121	0.060308	0.150*	
H7A2	0.486747	0.354513	0.082492	0.150*	
H7A3	0.443885	0.326135	0.011349	0.150*	
C7B	0.6055 (6)	0.2824 (2)	0.1752 (3)	0.110 (2)	
H7B1	0.708755	0.283359	0.169304	0.164*	
H7B2	0.592127	0.254428	0.198026	0.164*	
H7B3	0.592894	0.307561	0.204499	0.164*	
C12A	0.6159 (6)	0.15410 (19)	−0.1381 (3)	0.0979 (17)	
H12A	0.702796	0.144134	−0.098918	0.147*	
H12B	0.641932	0.182432	−0.156237	0.147*	
H12C	0.593787	0.131693	−0.175486	0.147*	
C12B	0.4445 (7)	0.11789 (15)	−0.0729 (3)	0.0946 (17)	
H12D	0.543013	0.107441	−0.041676	0.142*	
H12E	0.399218	0.094643	−0.107233	0.142*	
H12F	0.374442	0.124680	−0.045039	0.142*	
C14A	0.0443 (5)	0.17299 (15)	−0.2336 (2)	0.0746 (13)	
H14A	−0.054456	0.172069	−0.223149	0.112*	
H14B	0.056985	0.145700	−0.258362	0.112*	
H14C	0.046527	0.198861	−0.263348	0.112*	
Ni2	0.05959 (6)	0.05839 (2)	0.25539 (3)	0.03924 (17)	
N21	−0.1278 (3)	0.05912 (10)	0.17423 (15)	0.0458 (7)	
H21	−0.104191	0.078124	0.137427	0.055*	
C22	−0.2528 (5)	0.08287 (15)	0.1966 (2)	0.0635 (11)	
H22A	−0.355426	0.073191	0.166936	0.076*	
H22B	−0.244200	0.115468	0.191214	0.076*	
C23	−0.2330 (4)	0.07126 (14)	0.2733 (2)	0.0600 (11)	
H23A	−0.302809	0.089655	0.291809	0.072*	
H23B	−0.257880	0.039484	0.277655	0.072*	
N24	−0.0664 (3)	0.08048 (10)	0.31420 (15)	0.0470 (8)	
H24	−0.055655	0.113641	0.314496	0.056*	
C25	−0.0287 (5)	0.06706 (14)	0.3911 (2)	0.0573 (10)	
H25	−0.045248	0.034216	0.393331	0.069*	
C26	0.1421 (5)	0.07703 (14)	0.42877 (19)	0.0578 (11)	
H26A	0.159090	0.073180	0.479875	0.069*	
H26B	0.161403	0.108758	0.420735	0.069*	
C27	0.2636 (5)	0.04865 (13)	0.40731 (19)	0.0531 (10)	
N28	0.2554 (3)	0.06168 (10)	0.33122 (14)	0.0441 (7)	
H28	0.283461	0.093988	0.334118	0.053*	
C29	0.3799 (4)	0.03908 (15)	0.3064 (2)	0.0588 (11)	
H29A	0.481942	0.051537	0.331708	0.071*	
H29B	0.381236	0.006718	0.316162	0.071*	
C30	0.3462 (4)	0.04692 (14)	0.2288 (2)	0.0557 (10)	
H30A	0.409650	0.026684	0.209428	0.067*	
H30B	0.372762	0.077978	0.220239	0.067*	
N31	0.1765 (3)	0.03858 (9)	0.19211 (14)	0.0421 (7)	
H31	0.148791	0.060096	0.151995	0.050*	
C32	0.1288 (5)	−0.00772 (12)	0.15832 (19)	0.0483 (9)	
C33	−0.0400 (4)	−0.00298 (13)	0.11006 (19)	0.0515 (10)	
H33A	−0.038622	0.018142	0.072057	0.062*	
H33B	−0.071470	−0.032286	0.087650	0.062*	
C34	−0.1677 (4)	0.01252 (13)	0.1419 (2)	0.0518 (10)	
H34	−0.173451	−0.008818	0.179632	0.062*	
C25A	−0.1327 (6)	0.09093 (19)	0.4291 (2)	0.0884 (16)	
H25A	−0.238226	0.079776	0.410899	0.133*	
H25B	−0.131361	0.123076	0.420719	0.133*	
H25C	−0.094300	0.085053	0.479626	0.133*	
C27A	0.4261 (5)	0.06038 (16)	0.4582 (2)	0.0758 (13)	
H27A	0.503880	0.041184	0.447818	0.114*	
H27B	0.425999	0.055662	0.506881	0.114*	
H27C	0.450018	0.091620	0.451699	0.114*	
C27B	0.2314 (5)	−0.00229 (13)	0.4118 (2)	0.0705 (13)	
H27D	0.317339	−0.019414	0.404701	0.106*	
H27E	0.136392	−0.010190	0.375432	0.106*	
H27F	0.220133	−0.009180	0.458190	0.106*	
C32A	0.1411 (5)	−0.04402 (13)	0.2156 (2)	0.0653 (12)	
H32A	0.248832	−0.047298	0.243634	0.098*	
H32B	0.103155	−0.072465	0.192932	0.098*	
H32C	0.079130	−0.035147	0.246139	0.098*	
C32B	0.2321 (5)	−0.02093 (15)	0.1112 (2)	0.0683 (12)	
H32D	0.238820	0.004179	0.080827	0.102*	
H32E	0.186955	−0.046664	0.082207	0.102*	
H32F	0.335303	−0.028603	0.141077	0.102*	
C34A	−0.3247 (5)	0.01198 (16)	0.0823 (2)	0.0732 (13)	
H34A	−0.409332	0.015369	0.103082	0.110*	
H34B	−0.335778	−0.016353	0.056878	0.110*	
H34C	−0.327140	0.036600	0.049674	0.110*	
Cl1	0.04648 (14)	0.12152 (3)	0.02020 (5)	0.0627 (3)	
O11	0.0208 (4)	0.11563 (11)	−0.05487 (15)	0.0871 (10)	
O12	0.1777 (4)	0.09572 (13)	0.05975 (19)	0.1046 (12)	
O13	−0.0888 (4)	0.10628 (11)	0.03875 (16)	0.0824 (9)	
O14	0.0730 (6)	0.16810 (10)	0.03803 (18)	0.1168 (15)	
Cl2	−0.30063 (16)	−0.05995 (4)	0.32668 (7)	0.0744 (3)	
O21	−0.317 (2)	−0.0287 (6)	0.3798 (9)	0.108 (5)	0.5
O22	−0.4040 (14)	−0.0460 (6)	0.2612 (7)	0.136 (6)	0.5
O23	−0.305 (3)	−0.1036 (4)	0.3459 (11)	0.167 (7)	0.5
O24	−0.1491 (10)	−0.0529 (4)	0.3129 (7)	0.125 (3)	0.5
O25	−0.279 (3)	−0.0260 (6)	0.3745 (12)	0.149 (8)	0.5
O26	−0.319 (3)	−0.0460 (6)	0.2625 (9)	0.224 (10)	0.5
O27	−0.204 (3)	−0.0931 (8)	0.3517 (16)	0.261 (11)	0.5
O28	−0.4420 (17)	−0.0831 (5)	0.3231 (8)	0.194 (6)	0.5
Cl3	0.06049 (14)	0.18122 (3)	0.22458 (6)	0.0676 (3)	
O31	0.1373 (4)	0.14274 (10)	0.20864 (17)	0.0921 (11)	
O32	0.1437 (6)	0.22121 (11)	0.2232 (2)	0.1241 (15)	
O33	0.0440 (6)	0.17559 (12)	0.2931 (2)	0.1381 (18)	
O34	−0.0876 (5)	0.18279 (19)	0.1739 (3)	0.169 (2)	
Cl4A	0.5256 (7)	0.3146 (2)	−0.1429 (3)	0.0736 (9)	0.650 (8)
O41	0.5146 (12)	0.3523 (3)	−0.1872 (5)	0.132 (5)	0.650 (8)
O42	0.6606 (14)	0.3181 (5)	−0.0781 (5)	0.126 (4)	0.650 (8)
O43	0.5089 (18)	0.2745 (4)	−0.1693 (6)	0.192 (5)	0.650 (8)
O44	0.3937 (8)	0.3124 (3)	−0.1183 (3)	0.138 (3)	0.650 (8)
Cl4B	0.5700 (13)	0.3152 (4)	−0.1545 (7)	0.0736 (9)	0.350 (8)
O45	0.489 (2)	0.3555 (6)	−0.1438 (10)	0.142 (9)	0.350 (8)
O46	0.661 (3)	0.2972 (9)	−0.1007 (12)	0.136 (8)	0.350 (8)
O47	0.657 (2)	0.3202 (5)	−0.1969 (8)	0.164 (7)	0.350 (8)
O48	0.4912 (13)	0.2824 (8)	−0.2072 (9)	0.148 (7)	0.350 (8)
OW	0.4397 (5)	0.16490 (14)	0.1461 (2)	0.0980 (11)	
HW1	0.475 (6)	0.165 (2)	0.1897 (7)	0.147*	
HW2	0.354 (4)	0.152 (2)	0.134 (3)	0.147*	

### (5,7,7,12,12,14-Hexamethyl-1,4,8,11-tetraazacyclotetradecane)nickel(II) bis(perchlorate) hemihydrate (I) Atomic displacement parameters (Å^2^)

**Table:**

	*U*^11^	*U*^22^	*U*^33^	*U*^12^	*U*^13^	*U*^23^
Ni1	0.0521 (4)	0.0436 (4)	0.0405 (3)	0.0037 (3)	0.0125 (3)	0.0048 (3)
N1	0.059 (2)	0.0434 (18)	0.0428 (17)	−0.0001 (15)	0.0138 (15)	0.0058 (14)
C2	0.060 (3)	0.073 (3)	0.051 (2)	0.010 (2)	0.011 (2)	0.005 (2)
C3	0.067 (3)	0.076 (3)	0.056 (3)	0.020 (2)	0.016 (2)	0.005 (2)
N4	0.056 (2)	0.0442 (18)	0.0462 (18)	0.0025 (15)	0.0161 (16)	0.0039 (14)
C5	0.087 (3)	0.052 (2)	0.056 (3)	0.000 (2)	0.029 (2)	−0.003 (2)
C6	0.093 (4)	0.059 (3)	0.052 (3)	−0.006 (2)	0.029 (3)	−0.006 (2)
C7	0.075 (3)	0.070 (3)	0.061 (3)	−0.017 (2)	0.023 (2)	−0.011 (2)
N8	0.061 (2)	0.054 (2)	0.0459 (19)	−0.0004 (17)	0.0093 (16)	0.0042 (15)
C9	0.054 (3)	0.110 (4)	0.068 (3)	0.011 (3)	−0.005 (2)	−0.003 (3)
C10	0.072 (3)	0.104 (4)	0.077 (3)	0.037 (3)	0.006 (3)	−0.005 (3)
N11	0.056 (2)	0.0523 (19)	0.0514 (19)	0.0105 (16)	0.0159 (16)	0.0073 (15)
C12	0.072 (3)	0.052 (3)	0.071 (3)	0.008 (2)	0.028 (2)	−0.004 (2)
C13	0.090 (4)	0.064 (3)	0.057 (3)	−0.012 (3)	0.028 (3)	−0.011 (2)
C14	0.078 (3)	0.047 (2)	0.053 (2)	−0.004 (2)	0.020 (2)	−0.0026 (19)
C5A	0.106 (4)	0.095 (4)	0.072 (3)	0.024 (3)	0.043 (3)	−0.006 (3)
C7A	0.138 (5)	0.068 (3)	0.113 (4)	−0.029 (3)	0.067 (4)	−0.008 (3)
C7B	0.094 (4)	0.152 (6)	0.072 (3)	−0.035 (4)	0.008 (3)	−0.036 (4)
C12A	0.101 (4)	0.101 (4)	0.104 (4)	0.023 (3)	0.051 (3)	−0.015 (3)
C12B	0.120 (5)	0.057 (3)	0.107 (4)	0.014 (3)	0.034 (4)	0.021 (3)
C14A	0.087 (4)	0.070 (3)	0.058 (3)	−0.011 (3)	0.008 (3)	−0.010 (2)
Ni2	0.0378 (3)	0.0382 (3)	0.0413 (3)	0.0012 (3)	0.0109 (3)	0.0022 (3)
N21	0.0398 (17)	0.0485 (18)	0.0473 (17)	0.0015 (15)	0.0096 (14)	0.0041 (14)
C22	0.049 (3)	0.066 (3)	0.070 (3)	0.008 (2)	0.009 (2)	−0.005 (2)
C23	0.041 (2)	0.067 (3)	0.075 (3)	0.004 (2)	0.022 (2)	−0.011 (2)
N24	0.0465 (19)	0.0460 (18)	0.0502 (18)	0.0002 (15)	0.0169 (15)	−0.0003 (14)
C25	0.060 (3)	0.065 (3)	0.053 (2)	0.000 (2)	0.027 (2)	0.005 (2)
C26	0.071 (3)	0.062 (3)	0.041 (2)	−0.001 (2)	0.016 (2)	0.0021 (19)
C27	0.052 (2)	0.058 (3)	0.045 (2)	0.003 (2)	0.0084 (19)	0.0082 (19)
N28	0.0449 (18)	0.0434 (17)	0.0436 (17)	0.0034 (14)	0.0122 (14)	0.0014 (14)
C29	0.043 (2)	0.071 (3)	0.060 (3)	0.002 (2)	0.011 (2)	−0.005 (2)
C30	0.042 (2)	0.065 (3)	0.062 (3)	−0.004 (2)	0.018 (2)	−0.008 (2)
N31	0.0437 (18)	0.0418 (17)	0.0420 (16)	−0.0031 (14)	0.0145 (14)	−0.0004 (13)
C32	0.059 (3)	0.040 (2)	0.049 (2)	−0.0022 (19)	0.0186 (19)	−0.0043 (17)
C33	0.059 (3)	0.047 (2)	0.047 (2)	−0.008 (2)	0.013 (2)	−0.0030 (18)
C34	0.052 (2)	0.050 (2)	0.051 (2)	−0.0119 (19)	0.0110 (19)	0.0015 (18)
C25A	0.084 (4)	0.122 (4)	0.072 (3)	0.006 (3)	0.042 (3)	−0.011 (3)
C27A	0.068 (3)	0.097 (4)	0.050 (2)	0.003 (3)	−0.001 (2)	0.002 (2)
C27B	0.080 (3)	0.059 (3)	0.069 (3)	0.008 (2)	0.015 (2)	0.021 (2)
C32A	0.079 (3)	0.045 (2)	0.069 (3)	0.002 (2)	0.016 (2)	0.009 (2)
C32B	0.072 (3)	0.066 (3)	0.071 (3)	0.004 (2)	0.027 (2)	−0.020 (2)
C34A	0.053 (3)	0.084 (3)	0.072 (3)	−0.013 (2)	0.003 (2)	−0.012 (2)
Cl1	0.0884 (8)	0.0480 (6)	0.0518 (6)	−0.0145 (6)	0.0208 (6)	0.0056 (5)
O11	0.126 (3)	0.090 (2)	0.0525 (17)	−0.024 (2)	0.0374 (19)	−0.0008 (16)
O12	0.091 (2)	0.116 (3)	0.103 (3)	0.017 (2)	0.022 (2)	0.039 (2)
O13	0.099 (2)	0.083 (2)	0.075 (2)	−0.0148 (19)	0.0415 (19)	0.0139 (17)
O14	0.209 (4)	0.0520 (19)	0.088 (2)	−0.046 (2)	0.041 (3)	−0.0035 (17)
Cl2	0.0928 (9)	0.0517 (7)	0.0814 (8)	0.0066 (7)	0.0297 (7)	0.0028 (6)
O21	0.154 (11)	0.102 (8)	0.083 (6)	0.065 (9)	0.056 (7)	0.011 (5)
O22	0.101 (7)	0.162 (12)	0.103 (7)	0.053 (7)	−0.035 (6)	−0.021 (7)
O23	0.27 (2)	0.037 (4)	0.242 (16)	0.018 (9)	0.153 (17)	0.054 (6)
O24	0.068 (5)	0.160 (9)	0.166 (9)	0.007 (5)	0.061 (5)	0.016 (7)
O25	0.235 (18)	0.099 (8)	0.138 (10)	−0.069 (9)	0.094 (10)	−0.055 (7)
O26	0.47 (3)	0.128 (12)	0.122 (9)	−0.061 (18)	0.161 (15)	0.026 (8)
O27	0.250 (18)	0.193 (19)	0.33 (2)	0.157 (17)	0.07 (2)	0.041 (16)
O28	0.169 (10)	0.165 (12)	0.228 (14)	−0.083 (9)	0.027 (10)	0.033 (11)
Cl3	0.0846 (8)	0.0481 (6)	0.0707 (7)	0.0035 (6)	0.0235 (6)	0.0008 (5)
O31	0.133 (3)	0.0535 (18)	0.097 (2)	0.0242 (19)	0.045 (2)	0.0007 (17)
O32	0.192 (4)	0.053 (2)	0.142 (3)	−0.033 (2)	0.072 (3)	0.006 (2)
O33	0.263 (6)	0.084 (3)	0.106 (3)	−0.037 (3)	0.113 (3)	−0.024 (2)
O34	0.092 (3)	0.207 (5)	0.178 (5)	0.035 (3)	−0.005 (3)	0.019 (4)
Cl4A	0.083 (3)	0.0633 (8)	0.074 (2)	−0.008 (2)	0.0223 (15)	0.0158 (12)
O41	0.118 (7)	0.092 (6)	0.149 (8)	−0.043 (5)	−0.015 (6)	0.073 (6)
O42	0.086 (5)	0.210 (13)	0.075 (6)	0.005 (7)	0.010 (4)	0.020 (5)
O43	0.393 (16)	0.079 (5)	0.116 (8)	−0.050 (7)	0.094 (8)	−0.006 (6)
O44	0.093 (5)	0.223 (9)	0.097 (5)	−0.045 (5)	0.024 (4)	0.011 (5)
Cl4B	0.083 (3)	0.0633 (8)	0.074 (2)	−0.008 (2)	0.0223 (15)	0.0158 (12)
O45	0.140 (14)	0.098 (11)	0.158 (17)	0.061 (10)	−0.003 (12)	−0.026 (11)
O46	0.123 (12)	0.18 (2)	0.092 (11)	0.071 (13)	0.016 (9)	0.031 (11)
O47	0.273 (17)	0.138 (12)	0.129 (10)	−0.023 (11)	0.136 (12)	−0.011 (9)
O48	0.214 (15)	0.125 (14)	0.105 (12)	0.006 (9)	0.045 (10)	−0.055 (11)
OW	0.098 (3)	0.099 (3)	0.089 (2)	−0.011 (2)	0.015 (2)	0.019 (2)

### (5,7,7,12,12,14-Hexamethyl-1,4,8,11-tetraazacyclotetradecane)nickel(II) bis(perchlorate) hemihydrate (I) Geometric parameters (Å, º)

**Table:**

Ni1—N1	1.954 (3)	N24—C25	1.491 (5)
Ni1—N4	1.950 (3)	N24—H24	0.9800
Ni1—N8	1.949 (3)	C25—C26	1.511 (5)
Ni1—N11	1.956 (3)	C25—C25A	1.517 (5)
N1—C2	1.481 (5)	C25—H25	0.9800
N1—C14	1.508 (5)	C26—C27	1.520 (5)
N1—H1	0.9800	C26—H26A	0.9700
C2—C3	1.489 (5)	C26—H26B	0.9700
C2—H2A	0.9700	C27—N28	1.514 (4)
C2—H2B	0.9700	C27—C27B	1.533 (5)
C3—N4	1.482 (5)	C27—C27A	1.535 (5)
C3—H3A	0.9700	N28—C29	1.489 (5)
C3—H3B	0.9700	N28—H28	0.9800
N4—C5	1.496 (5)	C29—C30	1.474 (5)
N4—H4	0.9800	C29—H29A	0.9700
C5—C6	1.500 (6)	C29—H29B	0.9700
C5—C5A	1.524 (6)	C30—N31	1.490 (4)
C5—H5	0.9800	C30—H30A	0.9700
C6—C7	1.517 (6)	C30—H30B	0.9700
C6—H6A	0.9700	N31—C32	1.518 (4)
C6—H6B	0.9700	N31—H31	0.9800
C7—N8	1.504 (5)	C32—C32A	1.525 (5)
C7—C7A	1.532 (6)	C32—C32B	1.528 (5)
C7—C7B	1.547 (6)	C32—C33	1.529 (5)
N8—C9	1.475 (5)	C33—C34	1.517 (5)
N8—H8	0.9800	C33—H33A	0.9700
C9—C10	1.484 (6)	C33—H33B	0.9700
C9—H9A	0.9700	C34—C34A	1.534 (5)
C9—H9B	0.9700	C34—H34	0.9800
C10—N11	1.487 (5)	C25A—H25A	0.9600
C10—H10A	0.9700	C25A—H25B	0.9600
C10—H10B	0.9700	C25A—H25C	0.9600
N11—C12	1.515 (5)	C27A—H27A	0.9600
N11—H11	0.9800	C27A—H27B	0.9600
C12—C13	1.521 (6)	C27A—H27C	0.9600
C12—C12B	1.522 (6)	C27B—H27D	0.9600
C12—C12A	1.532 (6)	C27B—H27E	0.9600
C13—C14	1.495 (6)	C27B—H27F	0.9600
C13—H13A	0.9700	C32A—H32A	0.9600
C13—H13B	0.9700	C32A—H32B	0.9600
C14—C14A	1.527 (5)	C32A—H32C	0.9600
C14—H14	0.9800	C32B—H32D	0.9600
C5A—H5A1	0.9600	C32B—H32E	0.9600
C5A—H5A2	0.9600	C32B—H32F	0.9600
C5A—H5A3	0.9600	C34A—H34A	0.9600
C7A—H7A1	0.9600	C34A—H34B	0.9600
C7A—H7A2	0.9600	C34A—H34C	0.9600
C7A—H7A3	0.9600	Cl1—O14	1.416 (3)
C7B—H7B1	0.9600	Cl1—O12	1.417 (3)
C7B—H7B2	0.9600	Cl1—O11	1.425 (3)
C7B—H7B3	0.9600	Cl1—O13	1.428 (3)
C12A—H12A	0.9600	Cl2—O26	1.281 (13)
C12A—H12B	0.9600	Cl2—O27	1.299 (17)
C12A—H12C	0.9600	Cl2—O23	1.341 (10)
C12B—H12D	0.9600	Cl2—O25	1.342 (17)
C12B—H12E	0.9600	Cl2—O22	1.399 (11)
C12B—H12F	0.9600	Cl2—O28	1.414 (11)
C14A—H14A	0.9600	Cl2—O21	1.424 (17)
C14A—H14B	0.9600	Cl2—O24	1.466 (7)
C14A—H14C	0.9600	Cl3—O32	1.395 (3)
Ni2—N21	1.935 (3)	Cl3—O33	1.397 (4)
Ni2—N24	1.937 (3)	Cl3—O34	1.398 (4)
Ni2—N28	1.931 (3)	Cl3—O31	1.403 (3)
Ni2—N31	1.924 (3)	Cl4A—O43	1.279 (12)
Ni2—O31	2.799 (3)	Cl4A—O41	1.391 (9)
N21—C22	1.484 (5)	Cl4A—O44	1.394 (8)
N21—C34	1.507 (4)	Cl4A—O42	1.471 (11)
N21—H21	0.9800	Cl4B—O46	1.24 (3)
C22—C23	1.494 (5)	Cl4B—O47	1.300 (15)
C22—H22A	0.9700	Cl4B—O48	1.434 (19)
C22—H22B	0.9700	Cl4B—O45	1.44 (2)
C23—N24	1.489 (4)	O47—O48	1.82 (2)
C23—H23A	0.9700	OW—HW1	0.815 (10)
C23—H23B	0.9700	OW—HW2	0.821 (10)
			
N8—Ni1—N4	93.49 (14)	N24—C23—C22	107.6 (3)
N8—Ni1—N1	177.45 (13)	N24—C23—H23A	110.2
N4—Ni1—N1	86.74 (13)	C22—C23—H23A	110.2
N8—Ni1—N11	87.00 (14)	N24—C23—H23B	110.2
N4—Ni1—N11	177.59 (13)	C22—C23—H23B	110.2
N1—Ni1—N11	92.88 (13)	H23A—C23—H23B	108.5
C2—N1—C14	110.6 (3)	C23—N24—C25	112.7 (3)
C2—N1—Ni1	109.0 (2)	C23—N24—Ni2	106.3 (2)
C14—N1—Ni1	118.4 (2)	C25—N24—Ni2	120.5 (2)
C2—N1—H1	106.0	C23—N24—H24	105.3
C14—N1—H1	106.0	C25—N24—H24	105.3
Ni1—N1—H1	106.0	Ni2—N24—H24	105.3
N1—C2—C3	107.1 (3)	N24—C25—C26	109.6 (3)
N1—C2—H2A	110.3	N24—C25—C25A	112.2 (3)
C3—C2—H2A	110.3	C26—C25—C25A	110.1 (3)
N1—C2—H2B	110.3	N24—C25—H25	108.2
C3—C2—H2B	110.3	C26—C25—H25	108.2
H2A—C2—H2B	108.5	C25A—C25—H25	108.2
N4—C3—C2	108.3 (3)	C25—C26—C27	117.1 (3)
N4—C3—H3A	110.0	C25—C26—H26A	108.0
C2—C3—H3A	110.0	C27—C26—H26A	108.0
N4—C3—H3B	110.0	C25—C26—H26B	108.0
C2—C3—H3B	110.0	C27—C26—H26B	108.0
H3A—C3—H3B	108.4	H26A—C26—H26B	107.3
C3—N4—C5	110.6 (3)	N28—C27—C26	107.3 (3)
C3—N4—Ni1	107.1 (2)	N28—C27—C27B	110.3 (3)
C5—N4—Ni1	123.5 (3)	C26—C27—C27B	111.1 (3)
C3—N4—H4	104.7	N28—C27—C27A	110.0 (3)
C5—N4—H4	104.7	C26—C27—C27A	108.1 (3)
Ni1—N4—H4	104.7	C27B—C27—C27A	109.8 (3)
N4—C5—C6	111.0 (3)	C29—N28—C27	112.3 (3)
N4—C5—C5A	111.1 (4)	C29—N28—Ni2	108.7 (2)
C6—C5—C5A	110.7 (4)	C27—N28—Ni2	120.8 (2)
N4—C5—H5	108.0	C29—N28—H28	104.5
C6—C5—H5	108.0	C27—N28—H28	104.5
C5A—C5—H5	108.0	Ni2—N28—H28	104.5
C5—C6—C7	116.6 (4)	C30—C29—N28	108.5 (3)
C5—C6—H6A	108.1	C30—C29—H29A	110.0
C7—C6—H6A	108.1	N28—C29—H29A	110.0
C5—C6—H6B	108.1	C30—C29—H29B	110.0
C7—C6—H6B	108.1	N28—C29—H29B	110.0
H6A—C6—H6B	107.3	H29A—C29—H29B	108.4
N8—C7—C6	107.6 (3)	C29—C30—N31	109.9 (3)
N8—C7—C7A	110.2 (4)	C29—C30—H30A	109.7
C6—C7—C7A	111.8 (4)	N31—C30—H30A	109.7
N8—C7—C7B	109.5 (4)	C29—C30—H30B	109.7
C6—C7—C7B	107.0 (4)	N31—C30—H30B	109.7
C7A—C7—C7B	110.7 (4)	H30A—C30—H30B	108.2
C9—N8—C7	112.4 (3)	C30—N31—C32	118.1 (3)
C9—N8—Ni1	107.2 (2)	C30—N31—Ni2	107.8 (2)
C7—N8—Ni1	121.6 (3)	C32—N31—Ni2	114.5 (2)
C9—N8—H8	104.7	C30—N31—H31	105.0
C7—N8—H8	104.7	C32—N31—H31	105.0
Ni1—N8—H8	104.7	Ni2—N31—H31	105.0
N8—C9—C10	107.9 (4)	N31—C32—C32A	111.0 (3)
N8—C9—H9A	110.1	N31—C32—C32B	110.4 (3)
C10—C9—H9A	110.1	C32A—C32—C32B	109.6 (3)
N8—C9—H9B	110.1	N31—C32—C33	106.6 (3)
C10—C9—H9B	110.1	C32A—C32—C33	111.2 (3)
H9A—C9—H9B	108.4	C32B—C32—C33	108.0 (3)
C9—C10—N11	107.1 (4)	C34—C33—C32	119.7 (3)
C9—C10—H10A	110.3	C34—C33—H33A	107.4
N11—C10—H10A	110.3	C32—C33—H33A	107.4
C9—C10—H10B	110.3	C34—C33—H33B	107.4
N11—C10—H10B	110.3	C32—C33—H33B	107.4
H10A—C10—H10B	108.6	H33A—C33—H33B	106.9
C10—N11—C12	112.4 (3)	N21—C34—C33	109.4 (3)
C10—N11—Ni1	107.5 (3)	N21—C34—C34A	112.2 (3)
C12—N11—Ni1	123.5 (3)	C33—C34—C34A	108.2 (3)
C10—N11—H11	103.7	N21—C34—H34	109.0
C12—N11—H11	103.7	C33—C34—H34	109.0
Ni1—N11—H11	103.7	C34A—C34—H34	109.0
N11—C12—C13	108.4 (3)	C25—C25A—H25A	109.5
N11—C12—C12B	110.3 (4)	C25—C25A—H25B	109.5
C13—C12—C12B	112.3 (4)	H25A—C25A—H25B	109.5
N11—C12—C12A	108.9 (4)	C25—C25A—H25C	109.5
C13—C12—C12A	107.2 (4)	H25A—C25A—H25C	109.5
C12B—C12—C12A	109.5 (4)	H25B—C25A—H25C	109.5
C14—C13—C12	115.9 (4)	C27—C27A—H27A	109.5
C14—C13—H13A	108.3	C27—C27A—H27B	109.5
C12—C13—H13A	108.3	H27A—C27A—H27B	109.5
C14—C13—H13B	108.3	C27—C27A—H27C	109.5
C12—C13—H13B	108.3	H27A—C27A—H27C	109.5
H13A—C13—H13B	107.4	H27B—C27A—H27C	109.5
C13—C14—N1	109.0 (3)	C27—C27B—H27D	109.5
C13—C14—C14A	110.7 (3)	C27—C27B—H27E	109.5
N1—C14—C14A	112.4 (3)	H27D—C27B—H27E	109.5
C13—C14—H14	108.2	C27—C27B—H27F	109.5
N1—C14—H14	108.2	H27D—C27B—H27F	109.5
C14A—C14—H14	108.2	H27E—C27B—H27F	109.5
C5—C5A—H5A1	109.5	C32—C32A—H32A	109.5
C5—C5A—H5A2	109.5	C32—C32A—H32B	109.5
H5A1—C5A—H5A2	109.5	H32A—C32A—H32B	109.5
C5—C5A—H5A3	109.5	C32—C32A—H32C	109.5
H5A1—C5A—H5A3	109.5	H32A—C32A—H32C	109.5
H5A2—C5A—H5A3	109.5	H32B—C32A—H32C	109.5
C7—C7A—H7A1	109.5	C32—C32B—H32D	109.5
C7—C7A—H7A2	109.5	C32—C32B—H32E	109.5
H7A1—C7A—H7A2	109.5	H32D—C32B—H32E	109.5
C7—C7A—H7A3	109.5	C32—C32B—H32F	109.5
H7A1—C7A—H7A3	109.5	H32D—C32B—H32F	109.5
H7A2—C7A—H7A3	109.5	H32E—C32B—H32F	109.5
C7—C7B—H7B1	109.5	C34—C34A—H34A	109.5
C7—C7B—H7B2	109.5	C34—C34A—H34B	109.5
H7B1—C7B—H7B2	109.5	H34A—C34A—H34B	109.5
C7—C7B—H7B3	109.5	C34—C34A—H34C	109.5
H7B1—C7B—H7B3	109.5	H34A—C34A—H34C	109.5
H7B2—C7B—H7B3	109.5	H34B—C34A—H34C	109.5
C12—C12A—H12A	109.5	O14—Cl1—O12	109.4 (3)
C12—C12A—H12B	109.5	O14—Cl1—O11	109.59 (19)
H12A—C12A—H12B	109.5	O12—Cl1—O11	110.7 (2)
C12—C12A—H12C	109.5	O14—Cl1—O13	109.5 (2)
H12A—C12A—H12C	109.5	O12—Cl1—O13	108.4 (2)
H12B—C12A—H12C	109.5	O11—Cl1—O13	109.2 (2)
C12—C12B—H12D	109.5	O26—Cl2—O27	119.2 (16)
C12—C12B—H12E	109.5	O26—Cl2—O25	113.0 (13)
H12D—C12B—H12E	109.5	O27—Cl2—O25	110.6 (16)
C12—C12B—H12F	109.5	O23—Cl2—O22	118.0 (12)
H12D—C12B—H12F	109.5	O26—Cl2—O28	104.5 (12)
H12E—C12B—H12F	109.5	O27—Cl2—O28	98.1 (12)
C14—C14A—H14A	109.5	O25—Cl2—O28	109.8 (12)
C14—C14A—H14B	109.5	O23—Cl2—O21	113.5 (11)
H14A—C14A—H14B	109.5	O22—Cl2—O21	107.2 (9)
C14—C14A—H14C	109.5	O23—Cl2—O24	106.9 (8)
H14A—C14A—H14C	109.5	O22—Cl2—O24	100.8 (7)
H14B—C14A—H14C	109.5	O21—Cl2—O24	109.7 (10)
N31—Ni2—N28	88.24 (12)	O32—Cl3—O33	108.9 (2)
N31—Ni2—N21	88.72 (12)	O32—Cl3—O34	110.8 (3)
N28—Ni2—N21	174.45 (13)	O33—Cl3—O34	109.8 (3)
N31—Ni2—N24	176.44 (12)	O32—Cl3—O31	112.3 (3)
N28—Ni2—N24	94.58 (12)	O33—Cl3—O31	108.1 (2)
N21—Ni2—N24	88.30 (13)	O34—Cl3—O31	106.8 (3)
N31—Ni2—O31	80.07 (11)	Cl3—O31—Ni2	117.4 (2)
N28—Ni2—O31	87.20 (12)	O43—Cl4A—O41	120.3 (7)
N21—Ni2—O31	87.70 (12)	O43—Cl4A—O44	94.8 (8)
N24—Ni2—O31	97.88 (11)	O41—Cl4A—O44	109.8 (7)
C22—N21—C34	116.5 (3)	O43—Cl4A—O42	112.3 (9)
C22—N21—Ni2	107.8 (2)	O41—Cl4A—O42	111.7 (8)
C34—N21—Ni2	111.9 (2)	O44—Cl4A—O42	105.5 (6)
C22—N21—H21	106.7	O46—Cl4B—O47	102.8 (18)
C34—N21—H21	106.7	O46—Cl4B—O48	112.5 (17)
Ni2—N21—H21	106.7	O47—Cl4B—O48	83.1 (11)
N21—C22—C23	107.9 (3)	O46—Cl4B—O45	117.4 (15)
N21—C22—H22A	110.1	O47—Cl4B—O45	114.7 (14)
C23—C22—H22A	110.1	O48—Cl4B—O45	119.9 (13)
N21—C22—H22B	110.1	Cl4B—O47—O48	51.6 (9)
C23—C22—H22B	110.1	Cl4B—O48—O47	45.3 (6)
H22A—C22—H22B	108.4	HW1—OW—HW2	110 (2)
			
C14—N1—C2—C3	167.3 (3)	C22—C23—N24—Ni2	−41.8 (3)
Ni1—N1—C2—C3	35.5 (4)	C23—N24—C25—C26	179.5 (3)
N1—C2—C3—N4	−51.0 (4)	Ni2—N24—C25—C26	52.6 (4)
C2—C3—N4—C5	178.8 (3)	C23—N24—C25—C25A	−57.8 (4)
C2—C3—N4—Ni1	41.8 (4)	Ni2—N24—C25—C25A	175.3 (3)
C3—N4—C5—C6	−174.3 (3)	N24—C25—C26—C27	−67.9 (4)
Ni1—N4—C5—C6	−45.6 (4)	C25A—C25—C26—C27	168.2 (4)
C3—N4—C5—C5A	62.1 (4)	C25—C26—C27—N28	68.2 (4)
Ni1—N4—C5—C5A	−169.2 (3)	C25—C26—C27—C27B	−52.5 (5)
N4—C5—C6—C7	64.8 (5)	C25—C26—C27—C27A	−173.2 (3)
C5A—C5—C6—C7	−171.4 (4)	C26—C27—N28—C29	174.7 (3)
C5—C6—C7—N8	−69.8 (5)	C27B—C27—N28—C29	−64.1 (4)
C5—C6—C7—C7A	51.2 (5)	C27A—C27—N28—C29	57.3 (4)
C5—C6—C7—C7B	172.6 (4)	C26—C27—N28—Ni2	−55.0 (4)
C6—C7—N8—C9	−174.5 (4)	C27B—C27—N28—Ni2	66.2 (4)
C7A—C7—N8—C9	63.4 (5)	C27A—C27—N28—Ni2	−172.4 (3)
C7B—C7—N8—C9	−58.7 (5)	C27—N28—C29—C30	169.9 (3)
C6—C7—N8—Ni1	56.4 (4)	Ni2—N28—C29—C30	33.7 (4)
C7A—C7—N8—Ni1	−65.7 (5)	N28—C29—C30—N31	−45.5 (4)
C7B—C7—N8—Ni1	172.3 (3)	C29—C30—N31—C32	−96.4 (4)
C7—N8—C9—C10	−177.3 (4)	C29—C30—N31—Ni2	35.3 (4)
Ni1—N8—C9—C10	−41.0 (4)	C30—N31—C32—C32A	72.3 (4)
N8—C9—C10—N11	53.2 (5)	Ni2—N31—C32—C32A	−56.4 (4)
C9—C10—N11—C12	−178.2 (4)	C30—N31—C32—C32B	−49.4 (4)
C9—C10—N11—Ni1	−39.1 (5)	Ni2—N31—C32—C32B	−178.1 (2)
C10—N11—C12—C13	−179.4 (4)	C30—N31—C32—C33	−166.5 (3)
Ni1—N11—C12—C13	49.1 (4)	Ni2—N31—C32—C33	64.9 (3)
C10—N11—C12—C12B	57.2 (5)	N31—C32—C33—C34	−58.1 (4)
Ni1—N11—C12—C12B	−74.3 (4)	C32A—C32—C33—C34	63.1 (4)
C10—N11—C12—C12A	−63.1 (5)	C32B—C32—C33—C34	−176.6 (3)
Ni1—N11—C12—C12A	165.4 (3)	C22—N21—C34—C33	169.8 (3)
N11—C12—C13—C14	−64.9 (5)	Ni2—N21—C34—C33	−65.5 (3)
C12B—C12—C13—C14	57.3 (5)	C22—N21—C34—C34A	49.7 (4)
C12A—C12—C13—C14	177.7 (4)	Ni2—N21—C34—C34A	174.4 (3)
C12—C13—C14—N1	72.6 (4)	C32—C33—C34—N21	60.2 (4)
C12—C13—C14—C14A	−163.3 (4)	C32—C33—C34—C34A	−177.4 (3)
C2—N1—C14—C13	172.6 (3)	O32—Cl3—O31—Ni2	158.2 (2)
Ni1—N1—C14—C13	−60.6 (4)	O33—Cl3—O31—Ni2	38.0 (3)
C2—N1—C14—C14A	49.5 (4)	O34—Cl3—O31—Ni2	−80.2 (3)
Ni1—N1—C14—C14A	176.2 (3)	O46—Cl4B—O47—O48	111.6 (17)
C34—N21—C22—C23	91.2 (4)	O45—Cl4B—O47—O48	−119.9 (16)
Ni2—N21—C22—C23	−35.6 (4)	O46—Cl4B—O48—O47	−101.1 (19)
N21—C22—C23—N24	51.4 (4)	O45—Cl4B—O48—O47	114.6 (17)
C22—C23—N24—C25	−175.9 (3)		

### (5,7,7,12,12,14-Hexamethyl-1,4,8,11-tetraazacyclotetradecane)nickel(II) bis(perchlorate) hemihydrate (I) Hydrogen-bond geometry (Å, º)

**Table:**

*D*—H···*A*	*D*—H	H···*A*	*D*···*A*	*D*—H···*A*
N1—H1···O44	0.98	2.61	3.279 (9)	126
N4—H4···O14	0.98	2.02	2.941 (4)	157
N8—H8···O*W*	0.98	1.99	2.965 (5)	175
N11—H11···O43	0.98	2.11	3.028 (10)	155
N11—H11···O46	0.98	2.45	3.36 (3)	155
N21—H21···O13	0.98	2.14	3.093 (4)	165
N24—H24···O33	0.98	2.12	3.033 (5)	154
N28—H28···O45^i^	0.98	2.30	3.146 (16)	144
N31—H31···O12	0.98	2.16	3.083 (4)	156
O*W*—H*W*1···O41^i^	0.82 (1)	2.38 (2)	3.162 (11)	162 (5)
O*W*—H*W*1···O47^i^	0.82 (1)	2.37 (3)	3.139 (18)	157 (5)
O*W*—H*W*2···O12	0.82 (1)	2.45 (3)	3.181 (6)	149 (6)

Symmetry code: (i) *x*, −*y*+1/2, *z*+1/2.

### (5,7,7,12,12,14-Hexamethyl-1,4,8,11-tetraazacyclotetradecane)nickel(II) dibromide trihydrate (II) Crystal data

**Table:**

[Ni(C_16_H_36_N_4_)]Br_2_·3H_2_O	*D*_x_ = 1.549 Mg m^−^^3^*D*_m_ = 1.530 (3) Mg m^−^^3^*D*_m_ measured by Flotation in chloroform/carbon tetrachloride mixtures
*M**_r_* = 557.06	Mo *K*α radiation, λ = 0.7107 Å
Orthorhombic, *F**d**d*2	Cell parameters from 6096 reflections
*a* = 60.3649 (18) Å	θ = 0.4–27.5°
*b* = 19.8364 (9) Å	µ = 4.17 mm^−^^1^
*c* = 7.9773 (3) Å	*T* = 295 K
*V* = 9552.2 (6) Å^3^	Rod, yellow
*Z* = 16	0.37 × 0.15 × 0.10 mm
*F*(000) = 4608	

### (5,7,7,12,12,14-Hexamethyl-1,4,8,11-tetraazacyclotetradecane)nickel(II) dibromide trihydrate (II) Data collection

**Table:**

Enraf–Nonius KappaCCD diffractometer	5382 independent reflections
Radiation source: fine-focus sealed tube	4897 reflections with *I* > 2σ(*I*)
Graphite monochromator	*R*_int_ = 0.096
Detector resolution: 9 pixels mm^-1^	θ_max_ = 27.5°, θ_min_ = 2.2°
combination of ω and φ scans	*h* = −78→+78
Absorption correction: part of the refinement model (Δ*F*) (SCALEPACK; Otwinowski & Minor, 1997)	*k* = −25→+25
*T*_min_ = 0.34, *T*_max_ = 0.67	*l* = −10→+10
41142 measured reflections	

### (5,7,7,12,12,14-Hexamethyl-1,4,8,11-tetraazacyclotetradecane)nickel(II) dibromide trihydrate (II) Refinement

**Table:**

Refinement on *F*^2^	Secondary atom site location: difference Fourier map
Least-squares matrix: full	Hydrogen site location: mixed
*R*[*F*^2^ > 2σ(*F*^2^)] = 0.035	H atoms treated by a mixture of independent and constrained refinement
*wR*(*F*^2^) = 0.085	*w* = 1/[σ^2^(*F*_o_^2^) + (0.040*P*)^2^ + 35.*P*] where *P* = (*F*_o_^2^ + 2*F*_c_^2^)/3
*S* = 1.04	(Δ/σ)_max_ < 0.001
5382 reflections	Δρ_max_ = 0.88 e Å^−^^3^
253 parameters	Δρ_min_ = −0.56 e Å^−^^3^
4 restraints	Absolute structure: Twinning involves inversion,with Flack parameter corresponding to twin-fraction occupancies
Primary atom site location: heavy-atom method	

### (5,7,7,12,12,14-Hexamethyl-1,4,8,11-tetraazacyclotetradecane)nickel(II) dibromide trihydrate (II) Special details

**Table:**

Geometry. All esds (except the esd in the dihedral angle between two l.s. planes) are estimated using the full covariance matrix. The cell esds are taken into account individually in the estimation of esds in distances, angles and torsion angles; correlations between esds in cell parameters are only used when they are defined by crystal symmetry. An approximate (isotropic) treatment of cell esds is used for estimating esds involving l.s. planes.
Refinement. Refined as a 2-component perfect inversion twin.

### (5,7,7,12,12,14-Hexamethyl-1,4,8,11-tetraazacyclotetradecane)nickel(II) dibromide trihydrate (II) Fractional atomic coordinates and isotropic or equivalent isotropic displacement parameters (Å^2^)

**Table:**

	*x*	*y*	*z*	*U*_iso_*/*U*_eq_	
Br1	0.15971 (2)	0.23829 (3)	0.64516 (9)	0.0645 (2)	
Br2	0.20453 (2)	0.49097 (4)	0.45452 (7)	0.05935 (18)	
Ni	0.18885 (2)	0.41205 (3)	0.92870 (8)	0.03245 (13)	
N1	0.21367 (7)	0.4693 (2)	0.8794 (6)	0.0407 (10)	
H1	0.2135 (10)	0.476 (3)	0.779 (8)	0.041*	
C2	0.23414 (8)	0.4296 (3)	0.9187 (10)	0.0532 (14)	
H2A	0.237732	0.400071	0.825597	0.064*	
H2B	0.246536	0.459778	0.936969	0.064*	
C3	0.22999 (9)	0.3892 (3)	1.0713 (9)	0.0547 (15)	
H3A	0.228730	0.418374	1.168391	0.066*	
H3B	0.242077	0.357842	1.090118	0.066*	
N4	0.20894 (8)	0.3519 (2)	1.0439 (7)	0.0457 (11)	
H4	0.2107 (10)	0.322 (3)	0.979 (8)	0.046*	
C5	0.20145 (9)	0.3155 (3)	1.1970 (9)	0.0554 (14)	
H5	0.198881	0.348705	1.285962	0.067*	
C6	0.17993 (9)	0.2782 (3)	1.1644 (9)	0.0497 (13)	
H6A	0.176834	0.250079	1.261144	0.060*	
H6B	0.182237	0.248293	1.069725	0.060*	
C7	0.15935 (9)	0.3206 (3)	1.1287 (7)	0.0405 (11)	
N8	0.16302 (6)	0.3557 (2)	0.9622 (6)	0.0359 (9)	
H8	0.1650 (9)	0.328 (3)	0.884 (8)	0.036*	
C9	0.14319 (7)	0.3923 (2)	0.8992 (7)	0.0397 (11)	
H9A	0.136777	0.419087	0.988532	0.048*	
H9B	0.132137	0.360243	0.861173	0.048*	
C10	0.15002 (9)	0.4369 (3)	0.7573 (7)	0.0434 (12)	
H10A	0.152516	0.410081	0.657488	0.052*	
H10B	0.138332	0.469111	0.733394	0.052*	
N11	0.17079 (7)	0.4736 (2)	0.8034 (6)	0.0353 (8)	
H11	0.1758 (9)	0.480 (2)	0.700 (8)	0.035*	
C12	0.16868 (8)	0.5426 (2)	0.8824 (7)	0.0405 (11)	
C13	0.19218 (9)	0.5742 (3)	0.8846 (7)	0.0442 (12)	
H13A	0.196503	0.582262	0.769260	0.053*	
H13B	0.191057	0.617843	0.938697	0.053*	
C14	0.21089 (8)	0.5358 (3)	0.9689 (7)	0.0426 (11)	
H14	0.206878	0.527186	1.085942	0.051*	
C5A	0.21918 (13)	0.2649 (4)	1.2584 (14)	0.098 (4)	
H5A1	0.231669	0.289119	1.302265	0.147*	
H5A2	0.223883	0.237223	1.166271	0.147*	
H5A3	0.213000	0.236900	1.344699	0.147*	
C7A	0.13939 (11)	0.2730 (3)	1.1164 (9)	0.0587 (16)	
H7A1	0.140979	0.244693	1.019498	0.088*	
H7A2	0.126039	0.299016	1.106629	0.088*	
H7A3	0.138659	0.245500	1.215218	0.088*	
C7B	0.15541 (11)	0.3721 (3)	1.2670 (8)	0.0520 (14)	
H7B1	0.141211	0.392963	1.251117	0.078*	
H7B2	0.166781	0.405897	1.263101	0.078*	
H7B3	0.155744	0.349927	1.373886	0.078*	
C12A	0.15366 (12)	0.5887 (3)	0.7761 (10)	0.0657 (19)	
H12A	0.158590	0.588079	0.661714	0.098*	
H12B	0.154366	0.633877	0.818647	0.098*	
H12C	0.138661	0.572708	0.781776	0.098*	
C12B	0.15975 (10)	0.5374 (3)	1.0602 (8)	0.0493 (13)	
H12D	0.169109	0.508202	1.124882	0.074*	
H12E	0.144991	0.519431	1.057561	0.074*	
H12F	0.159513	0.581408	1.110415	0.074*	
C14A	0.23179 (10)	0.5796 (4)	0.9644 (12)	0.072 (2)	
H14A	0.243228	0.558536	1.029552	0.109*	
H14B	0.228530	0.623221	1.010142	0.109*	
H14C	0.236705	0.584401	0.850538	0.109*	
O1	0.20252 (16)	0.3394 (5)	0.6365 (12)	0.148 (3)	
O2	0.19992 (17)	0.1516 (4)	0.8373 (16)	0.167 (4)	
O3	0.22816 (10)	0.2518 (5)	0.801 (2)	0.248 (9)	

### (5,7,7,12,12,14-Hexamethyl-1,4,8,11-tetraazacyclotetradecane)nickel(II) dibromide trihydrate (II) Atomic displacement parameters (Å^2^)

**Table:**

	*U*^11^	*U*^22^	*U*^33^	*U*^12^	*U*^13^	*U*^23^
Br1	0.0804 (4)	0.0507 (3)	0.0625 (4)	−0.0051 (3)	−0.0021 (3)	−0.0088 (3)
Br2	0.0611 (3)	0.0760 (4)	0.0409 (3)	−0.0070 (3)	0.0056 (3)	0.0004 (3)
Ni	0.0272 (2)	0.0338 (3)	0.0364 (3)	−0.0001 (2)	−0.0010 (2)	−0.0003 (2)
N1	0.032 (2)	0.046 (2)	0.044 (3)	−0.0035 (17)	0.0034 (18)	0.001 (2)
C2	0.027 (2)	0.060 (3)	0.072 (4)	0.000 (2)	0.003 (3)	−0.002 (3)
C3	0.030 (2)	0.060 (3)	0.075 (4)	0.007 (2)	−0.010 (3)	0.001 (3)
N4	0.033 (2)	0.044 (3)	0.060 (3)	0.0056 (19)	−0.002 (2)	−0.004 (2)
C5	0.050 (3)	0.058 (3)	0.059 (4)	0.009 (2)	−0.008 (3)	0.018 (3)
C6	0.049 (3)	0.037 (3)	0.063 (4)	0.005 (2)	−0.002 (3)	0.010 (3)
C7	0.040 (3)	0.042 (3)	0.040 (3)	−0.003 (2)	−0.002 (2)	0.004 (2)
N8	0.0347 (19)	0.033 (2)	0.040 (2)	−0.0016 (15)	−0.0019 (18)	−0.0017 (17)
C9	0.030 (2)	0.041 (2)	0.048 (3)	−0.0043 (18)	−0.007 (2)	0.003 (2)
C10	0.044 (3)	0.043 (3)	0.044 (3)	−0.001 (2)	−0.014 (2)	0.002 (2)
N11	0.033 (2)	0.039 (2)	0.035 (2)	−0.0016 (16)	−0.0002 (17)	0.0037 (17)
C12	0.037 (2)	0.033 (2)	0.052 (3)	0.0020 (19)	−0.003 (2)	0.000 (2)
C13	0.046 (3)	0.036 (2)	0.051 (3)	−0.006 (2)	0.003 (2)	0.006 (2)
C14	0.039 (2)	0.045 (3)	0.043 (3)	−0.006 (2)	0.003 (2)	−0.005 (2)
C5A	0.067 (5)	0.088 (6)	0.140 (10)	0.017 (4)	−0.015 (5)	0.063 (6)
C7A	0.055 (3)	0.057 (4)	0.064 (4)	−0.018 (3)	0.002 (3)	0.012 (3)
C7B	0.059 (4)	0.058 (4)	0.040 (3)	0.006 (3)	0.004 (3)	0.002 (3)
C12A	0.063 (4)	0.045 (3)	0.089 (5)	0.005 (3)	−0.013 (4)	0.014 (3)
C12B	0.046 (3)	0.043 (3)	0.060 (4)	−0.003 (2)	0.016 (3)	−0.010 (3)
C14A	0.050 (3)	0.064 (4)	0.104 (6)	−0.017 (3)	−0.001 (4)	−0.015 (4)
O1	0.173 (8)	0.150 (8)	0.121 (8)	−0.029 (6)	0.007 (6)	−0.015 (6)
O2	0.192 (9)	0.094 (5)	0.216 (12)	0.025 (6)	−0.060 (8)	−0.008 (7)
O3	0.246 (15)	0.156 (9)	0.34 (2)	−0.014 (10)	0.074 (15)	−0.093 (12)

### (5,7,7,12,12,14-Hexamethyl-1,4,8,11-tetraazacyclotetradecane)nickel(II) dibromide trihydrate (II) Geometric parameters (Å, º)

**Table:**

Br1—O2	3.346 (9)	C10—H10A	0.9700
Br2—O1	3.341 (9)	C10—H10B	0.9700
Ni—N11	1.918 (4)	N11—C12	1.512 (6)
Ni—N1	1.921 (4)	N11—H11	0.89 (6)
Ni—N4	1.934 (5)	C12—C12B	1.521 (8)
Ni—N8	1.937 (4)	C12—C12A	1.541 (8)
Ni—O1	2.863 (10)	C12—C13	1.551 (7)
N1—C2	1.499 (7)	C13—C14	1.519 (7)
N1—C14	1.509 (7)	C13—H13A	0.9700
N1—H1	0.81 (6)	C13—H13B	0.9700
C2—C3	1.479 (10)	C14—C14A	1.531 (7)
C2—H2A	0.9700	C14—H14	0.9800
C2—H2B	0.9700	C5A—H5A1	0.9600
C3—N4	1.486 (7)	C5A—H5A2	0.9600
C3—H3A	0.9700	C5A—H5A3	0.9600
C3—H3B	0.9700	C7A—H7A1	0.9600
N4—C5	1.489 (8)	C7A—H7A2	0.9600
N4—H4	0.80 (6)	C7A—H7A3	0.9600
C5—C6	1.518 (8)	C7B—H7B1	0.9600
C5—C5A	1.548 (8)	C7B—H7B2	0.9600
C5—H5	0.9800	C7B—H7B3	0.9600
C6—C7	1.528 (7)	C12A—H12A	0.9600
C6—H6A	0.9700	C12A—H12B	0.9600
C6—H6B	0.9700	C12A—H12C	0.9600
C7—N8	1.515 (7)	C12B—H12D	0.9600
C7—C7B	1.522 (8)	C12B—H12E	0.9600
C7—C7A	1.535 (7)	C12B—H12F	0.9600
N8—C9	1.487 (6)	C14A—H14A	0.9600
N8—H8	0.83 (6)	C14A—H14B	0.9600
C9—C10	1.496 (7)	C14A—H14C	0.9600
C9—H9A	0.9700	O1—O3	2.671 (11)
C9—H9B	0.9700	O2—O3	2.635 (10)
C10—N11	1.495 (6)	O3—O3^i^	2.637 (12)
			
N11—Ni—N1	87.73 (19)	C10—N11—C12	118.1 (4)
N11—Ni—N4	175.6 (2)	C10—N11—Ni	107.2 (3)
N1—Ni—N4	88.5 (2)	C12—N11—Ni	114.0 (3)
N11—Ni—N8	88.93 (18)	C10—N11—H11	97 (3)
N1—Ni—N8	175.8 (2)	C12—N11—H11	107 (3)
N4—Ni—N8	94.79 (19)	Ni—N11—H11	112 (3)
N11—Ni—O1	93.4 (2)	N11—C12—C12B	110.9 (4)
N1—Ni—O1	84.6 (2)	N11—C12—C12A	110.9 (5)
N4—Ni—O1	84.0 (2)	C12B—C12—C12A	110.1 (5)
N8—Ni—O1	93.1 (2)	N11—C12—C13	107.1 (4)
C2—N1—C14	116.9 (5)	C12B—C12—C13	109.9 (4)
C2—N1—Ni	106.8 (3)	C12A—C12—C13	107.7 (4)
C14—N1—Ni	109.5 (3)	C14—C13—C12	118.9 (4)
C2—N1—H1	108 (4)	C14—C13—H13A	107.6
C14—N1—H1	108 (4)	C12—C13—H13A	107.6
Ni—N1—H1	107 (4)	C14—C13—H13B	107.6
C3—C2—N1	108.5 (5)	C12—C13—H13B	107.6
C3—C2—H2A	110.0	H13A—C13—H13B	107.0
N1—C2—H2A	110.0	N1—C14—C13	108.1 (4)
C3—C2—H2B	110.0	N1—C14—C14A	113.1 (5)
N1—C2—H2B	110.0	C13—C14—C14A	108.6 (5)
H2A—C2—H2B	108.4	N1—C14—H14	109.0
C2—C3—N4	107.0 (5)	C13—C14—H14	109.0
C2—C3—H3A	110.3	C14A—C14—H14	109.0
N4—C3—H3A	110.3	C5—C5A—H5A1	109.5
C2—C3—H3B	110.3	C5—C5A—H5A2	109.5
N4—C3—H3B	110.3	H5A1—C5A—H5A2	109.5
H3A—C3—H3B	108.6	C5—C5A—H5A3	109.5
C5—N4—C3	112.3 (5)	H5A1—C5A—H5A3	109.5
C5—N4—Ni	119.9 (3)	H5A2—C5A—H5A3	109.5
C3—N4—Ni	107.4 (4)	C7—C7A—H7A1	109.5
C5—N4—H4	102 (5)	C7—C7A—H7A2	109.5
C3—N4—H4	111 (4)	H7A1—C7A—H7A2	109.5
Ni—N4—H4	104 (5)	C7—C7A—H7A3	109.5
N4—C5—C6	110.9 (5)	H7A1—C7A—H7A3	109.5
N4—C5—C5A	111.4 (6)	H7A2—C7A—H7A3	109.5
C6—C5—C5A	109.2 (5)	C7—C7B—H7B1	109.5
N4—C5—H5	108.4	C7—C7B—H7B2	109.5
C6—C5—H5	108.4	H7B1—C7B—H7B2	109.5
C5A—C5—H5	108.4	C7—C7B—H7B3	109.5
C5—C6—C7	117.3 (4)	H7B1—C7B—H7B3	109.5
C5—C6—H6A	108.0	H7B2—C7B—H7B3	109.5
C7—C6—H6A	108.0	C12—C12A—H12A	109.5
C5—C6—H6B	108.0	C12—C12A—H12B	109.5
C7—C6—H6B	108.0	H12A—C12A—H12B	109.5
H6A—C6—H6B	107.2	C12—C12A—H12C	109.5
N8—C7—C7B	110.5 (4)	H12A—C12A—H12C	109.5
N8—C7—C6	107.3 (4)	H12B—C12A—H12C	109.5
C7B—C7—C6	111.2 (5)	C12—C12B—H12D	109.5
N8—C7—C7A	110.0 (4)	C12—C12B—H12E	109.5
C7B—C7—C7A	109.7 (5)	H12D—C12B—H12E	109.5
C6—C7—C7A	108.1 (4)	C12—C12B—H12F	109.5
C9—N8—C7	113.7 (4)	H12D—C12B—H12F	109.5
C9—N8—Ni	108.7 (3)	H12E—C12B—H12F	109.5
C7—N8—Ni	120.2 (3)	C14—C14A—H14A	109.5
C9—N8—H8	101 (4)	C14—C14A—H14B	109.5
C7—N8—H8	112 (4)	H14A—C14A—H14B	109.5
Ni—N8—H8	99 (4)	C14—C14A—H14C	109.5
N8—C9—C10	108.9 (4)	H14A—C14A—H14C	109.5
N8—C9—H9A	109.9	H14B—C14A—H14C	109.5
C10—C9—H9A	109.9	O3—O1—Ni	95.4 (4)
N8—C9—H9B	109.9	O3—O1—Br2	141.1 (4)
C10—C9—H9B	109.9	Ni—O1—Br2	84.9 (2)
H9A—C9—H9B	108.3	O3—O2—Br1	91.8 (3)
N11—C10—C9	109.4 (4)	O3—O2—Br2^ii^	134.3 (4)
N11—C10—H10A	109.8	Br1—O2—Br2^ii^	132.9 (3)
C9—C10—H10A	109.8	O3^i^—O3—O2	128.8 (6)
N11—C10—H10B	109.8	O3^i^—O3—O1	126.6 (5)
C9—C10—H10B	109.8	O2—O3—O1	99.8 (4)
H10A—C10—H10B	108.2		
			
C14—N1—C2—C3	84.7 (6)	C7—N8—C9—C10	167.4 (4)
Ni—N1—C2—C3	−38.3 (6)	Ni—N8—C9—C10	30.6 (5)
N1—C2—C3—N4	51.3 (6)	N8—C9—C10—N11	−45.2 (6)
C2—C3—N4—C5	−173.1 (5)	C9—C10—N11—C12	−92.8 (5)
C2—C3—N4—Ni	−39.2 (6)	C9—C10—N11—Ni	37.6 (5)
C3—N4—C5—C6	180.0 (5)	C10—N11—C12—C12B	70.9 (6)
Ni—N4—C5—C6	52.4 (6)	Ni—N11—C12—C12B	−56.3 (5)
C3—N4—C5—C5A	−58.2 (8)	C10—N11—C12—C12A	−51.8 (6)
Ni—N4—C5—C5A	174.2 (5)	Ni—N11—C12—C12A	−179.1 (4)
N4—C5—C6—C7	−66.1 (7)	C10—N11—C12—C13	−169.1 (4)
C5A—C5—C6—C7	170.9 (7)	Ni—N11—C12—C13	63.6 (5)
C5—C6—C7—N8	66.7 (7)	N11—C12—C13—C14	−55.5 (6)
C5—C6—C7—C7B	−54.3 (7)	C12B—C12—C13—C14	65.0 (6)
C5—C6—C7—C7A	−174.7 (6)	C12A—C12—C13—C14	−174.9 (5)
C7B—C7—N8—C9	−66.0 (5)	C2—N1—C14—C13	167.8 (4)
C6—C7—N8—C9	172.6 (4)	Ni—N1—C14—C13	−70.6 (5)
C7A—C7—N8—C9	55.2 (6)	C2—N1—C14—C14A	47.6 (8)
C7B—C7—N8—Ni	65.4 (5)	Ni—N1—C14—C14A	169.1 (5)
C6—C7—N8—Ni	−56.1 (5)	C12—C13—C14—N1	60.7 (6)
C7A—C7—N8—Ni	−173.4 (4)	C12—C13—C14—C14A	−176.2 (5)

Symmetry codes: (i) −*x*+1/2, −*y*+1/2, *z*; (ii) *x*, *y*−1/2, *z*+1/2.

### (5,7,7,12,12,14-Hexamethyl-1,4,8,11-tetraazacyclotetradecane)nickel(II) dibromide trihydrate (II) Hydrogen-bond geometry (Å, º)

**Table:**

*D*—H···*A*	*D*—H	H···*A*	*D*···*A*	*D*—H···*A*
N1—H1···Br2	0.81 (6)	2.66 (6)	3.461 (5)	169 (6)
N4—H4···O1	0.80 (6)	2.80 (7)	3.283 (11)	121 (5)
N4—H4···O3	0.80 (6)	2.25 (6)	3.008 (13)	159 (6)
N8—H8···Br1	0.83 (6)	2.63 (6)	3.444 (5)	164 (5)
N11—H11···Br2	0.89 (6)	2.63 (6)	3.466 (4)	159 (5)
